# Mapping of Lipidome Profile in Drug-Resistant Clinical Isolates of *Mycobacterium tuberculosis* Through Quali-Quantitative Liquid Chromatography-Mass Spectrometry Identifies Signature Lipids

**DOI:** 10.3390/life16060953

**Published:** 2026-06-05

**Authors:** Meenakshi Chugh, Saif Hameed, Umay Kulsum, Shivkumar Rashmi Mudliar, Jitendra Singh, Sarman Singh, Ashok Kumar Sah, Rabab H. Eilshaikh, Ranjay Kumar Choudhary, Zeeshan Fatima

**Affiliations:** 1 Amity Institute of Biotechnology, Amity University Haryana, Manesar, Gurugram 122413, India; meenakshijune1992@gmail.com (M.C.); saifhameed@yahoo.co.in (S.H.); 2 Amity Medical School, Amity University Haryana, Manesar, Gurugram 122413, India; 3 Department of Translational Medicine, All India Institute of Medical Sciences, Bhopal 462020, India; kulsum.umay@gmail.com (U.K.); jitendra.tmc@aiimsbhopal.edu.in (J.S.); 4 Department of Microbiology, All India Institute of Medical Sciences, Nagpur 441108, India; srashmim1@gmail.com; 5 Advanced Centre for Chronic and Rare Diseases, New Delhi 110068, India; sarman_singh@yahoo.com; 6 Department of Medical Laboratory Sciences, College of Applied and Health Sciences, A’ Sharqiyah University, Ibra 400, Oman; ashok.sah8@gmail.com (A.K.S.); ranjay.choudhary@asu.edu.om (R.K.C.)

**Keywords:** tuberculosis, *Mycobacterium tuberculosis*, drug resistance, drug-resistant tuberculosis, Liquid Chromatography-Mass Spectrometry, lipidomics

## Abstract

The complex lipid composition of *Mycobacterium tuberculosis* (MTB) plays a pivotal role in pathogenesis, immune evasion, and antimicrobial resistance. The continued surge in drug-resistant strains underscores the need for a deeper understanding of the molecular architecture underlying MTB pathogenesis and drug resistance. In this study, a comparative lipidomics approach was applied to explore the resistance-associated lipid alterations in drug-sensitive (DS), drug-resistant (DR), multidrug-resistant (MDR), and pre-extensively drug-resistant (PXDR) MTB clinical isolates. Lipids derived from whole bacilli (TL) and cell wall (CWL) extracts were separately analyzed using untargeted and targeted lipidomics approaches. Untargeted analysis revealed the abundance of fatty acyls, glycerolipids, glycerophospholipids, prenol lipids, polyketides, and saccharolipids, with distinct phenotypes and compartment-specific distributions. Notably, CWL extracts showed clearer resistance-associated separations relative to TL extracts. Targeted profiling further demonstrated enrichment of glycerolipids and PC and LPC lipids among drug-resistant isolates. Biomarker analysis identified discriminative lipid species in both extracts, albeit with greater discriminatory power in CWL. Overall, these findings elucidate coordinated and compartment-associated lipid alterations at the species level in different MTB clinical isolates. This will help to provide a valuable source for screening diagnostic biosignatures and intersecting biosynthetic pathways of mycobacterial lipids in the evolution of drug resistance in MTB for therapeutic interventions.

## 1. Introduction

*Mycobacterium tuberculosis* (MTB), the pathogen responsible for tuberculosis (TB), poses a persistent public health challenge globally. Despite effective antimicrobial treatments, TB continues to result in substantial morbidity and mortality, particularly in regions with a high prevalence of drug-resistant strains, viz., drug-resistant (DR), multi-drug-resistant (MDR), pre-extremely drug-resistant (PXDR), and extensively drug-resistant (XDR) phenotypes [1,2]. The emergence of resistant MTB strains complicates global TB management and eradication efforts. This necessitates a deeper and comprehensive understanding of the molecular mechanisms underlying MTB drug resistance, which is essential for developing more effective diagnostic, therapeutic, and preventive strategies [3].

Unique lipid metabolism and lipid composition of MTB have long been recognized as integral to its structural integrity, virulence, survival, and resistance to both drugs and host immune responses [4]. Alterations in lipid composition may contribute to the survival strategies of resistant strains, rendering lipidomic analysis a promising approach to uncover novel resistance mechanisms and potential therapeutic targets, yet it remains an underexplored area in MTB research [5]. The advent of advanced analytical techniques such as Liquid Chromatography-Mass Spectrometry (LC-MS) has revolutionized the field of lipidomics by enabling high-resolution profiling of lipid species with high sensitivity and specificity. It facilitates the identification of resistance-associated lipid biomarkers and enables differentiation between drug-sensitive and drug-resistant MTB strains [6,7,8].

Several studies have indicated that changes in the lipid composition of MTB can influence its susceptibility to first-line drugs, such as rifampicin and isoniazid, as well as second-line drugs used in the treatment of MDR and XDR-TB [6]. Lipids such as mycolic acids (MAs), phospholipids, glycolipids, and trehalose dimycolate (TDM) play pivotal roles in the bacteria’s ability to resist oxidative stress, modulate immune responses, and maintain membrane integrity under hostile conditions [4,5]. These lipids may also serve as key players in resistance mechanisms against drug action by altering cell membrane permeability, sequestering drugs, or affecting the drug’s target site. Thus, a comprehensive lipidomic profiling using LC-MS can provide a holistic view of how MTB adapts to its environment and survives under the selective pressure of antimicrobial agents [9].

Previously, significant differences in the three major lipid classes of MTB, viz. fatty acyl (FAs), glycerolipids (GLs), and glycerophospholipids (GPLs), have been reported. DR strains exhibited significantly higher levels of MAs, phosphatidylinositol mannosides (PIMs), phosphatidylinositol (PIs), cardiolipin (CL), and triacylglycerides (TAGs), all of which are vital for bacterial virulence and pathogenicity [9]. Building on current evidence, this study applies both untargeted and targeted LC-MS-based lipidomics to characterize lipidomic alterations correlated with varying drug resistance levels in clinical MTB isolates (DS, DR, MDR, and PXDR phenotypes). By analyzing total (TL) and cell wall lipid (CWL) extracts, the study aims to identify resistance-associated lipid signatures and potential biomarkers, thereby advancing our understanding of lipid-mediated resistance mechanisms. These insights could ultimately aid in the development of improved diagnostic tools and therapeutic interventions for TB-resistant forms. In essence, this work seeks to delineate distinct lipidomic patterns that may reveal critical aspects of pathogenicity and environmental adaptation in drug-sensitive and drug-resistant MTB isolates.

## 2. Materials and Methods

### 2.1. Materials and Chemicals

Middlebrook 7H9 broth and oleic acid albumin dextrose catalase (OADC) supplements were procured from BD Biosciences (San Jose, CA, USA). Tween-80 was sourced from Sigma-Aldrich (St. Louis, MO, USA), and glycerol, potassium chloride (KCl), and n-hexane were sourced from Fisher Scientific (Pittsburgh, PA, USA). CHROMASOLV™ grade—chloroform, methanol, acetonitrile (ACN), 2-propanol (IPA), formic acid, and ammonium acetate (AA) (Honeywell Fluka™) was obtained from Honeywell (Charlotte, NC, USA). SPLASH™ LIPIDOMIX™ mass spectrometry standard and lipid standards, including 1,2-dimyristoyl-sn-glycerol (DAG), 1,2-diheptadecanoyl-sn-glycero-3-phosphocholine (PC), 1-heptadecanoyl-2-hydroxy-sn-glycero-3-phosphocholine (LPC), 1,2-dimyristoyl-sn-glycero-3 phosphoethanolamine (PE), 1-heptadecanoyl-2-(9Z-tetradecenoyl)-sn glycero-3-phospho-(1′-myo-inositol (PI) and 1,2-dimyristoyl-sn-glycero-3-phospho-L serine (PS) were purchased from Avanti Polar Lipids ((Alabaster, AL, USA). Tripentadecanoin (TG) was obtained from Sigma-Aldrich. Teflon-capped glass vials were obtained from Borosil (Mumbai, Maharashtra, India), and Pasteur pipettes and Whatman No. 1 filter paper from Merck (Bengaluru, Karnataka, India).

### 2.2. Patient Sample Processing

Sputum samples were aseptically collected from treatment-naive patients with microbiologically confirmed pulmonary mycobacterial infections who had not yet started anti-tubercular or antibiotic therapy. Diagnosis was established by ZN staining, AFB culture, and GeneXpert [Sunnyvale, CA, USA] positivity. Mycobacteria were cultured from these samples under BSL-3 conditions and considered as clinical isolates. For each phenotype (DS, DR, MDR, and PXDR), four independent clinical isolates were included and analyzed as biological replicates.

### 2.3. Culture Conditions

DS, DR, MDR, and PXDR MTB clinical isolates were obtained from AIIMS, Bhopal, India, and used in this study. All strains were inoculated into 30 mL of Middlebrook 7H9 medium supplemented with 0.05% Tween-80, 0.2% glycerol, and 10% OADC. The cultures were incubated at 37 °C with constant agitation until the bacterial cells achieved the exponential growth phase (1.0 OD_600_ cells). The bacterial cells were carefully harvested to avoid cellular disruption and stored at −20 °C for further experimental procedures and analysis.

### 2.4. Protein Estimation

For lipid extraction, 1 OD_600_ cells (approximately 3 × 10^8^ cells/mL) were harvested, and total protein was estimated using a BCA protein assay. Cell lysate equivalent to 1 mg protein was used for both total lipid and cell wall lipid extraction across all clinical isolates to normalize potential differences in cell biomass [10].

### 2.5. Extraction of Total Lipids

TL from *Mycobacterium* cells was extracted using a modified Folch method, as described [8]. Briefly, the harvested cells were first homogenized in 1 mL aqueous solution by sonication for 3 min, with three cycles of 1 min intervals. Following homogenization, chloroform and methanol were added to each sample in a 1:2 ratio, and the mixture was then centrifuged at 2000 rpm and 4 °C for 10–15 min. Light-protected conditions were maintained during extraction. The supernatants were carefully transferred to new glass vials, and additional chloroform was added to achieve a final solvent ratio of 1:1 (chloroform:methanol). Efficient lipid recovery was facilitated by sonication-assisted homogenization and optimized solvent ratios. The solution was filtered through Whatman No. 1 filter paper to remove insoluble cellular debris prior to phase separation. The filtrates were then subjected to phase separation by the addition of 0.88% KCl, enabling efficient partitioning of lipids into the organic phase and removal of non-lipid contaminants. The lower organic phase containing the lipids was collected using a Pasteur pipette and transferred to glass vials with Teflon caps. The lipids were then dried under a stream of liquid nitrogen and stored at −20 °C until further analysis [9]. The reproducibility of lipid profiles across biological and technical replicates supports the consistency of the extraction procedure.

### 2.6. Extraction of Cell Wall Lipids

CWL were extracted using a modified Minnikin method, as described in [9]. Sequential solvent extraction using n-hexane, chloroform, and methanol was employed to recover lipid classes based on their differential solubility, where n-hexane preferentially extracted non-polar cell wall-associated lipids. Briefly, the harvested cells were mixed with 3 mL of n-hexane and incubated overnight in the dark at room temperature. The following day, the solutions were centrifuged at 2500 rpm for 10 min, and supernatants were carefully collected into a glass vial. Next, 3 mL of chloroform was added and incubated for 30 min at room temperature, followed by 3 mL of methanol for 2 h to ensure proper extraction. The upper organic phase enriched in cell wall-associated lipid species was collected in a new glass vial, dried using a gentle stream of nitrogen gas, and stored at −20 °C for future use [10].

### 2.7. Ultra-High-Performance Liquid Chromatography Coupled with Tandem Mass Spectrometry (UHP-LC-MS/MS)

Each biological replicate was analyzed in triplicate during UHP-LC-MS/MS acquisition for both untargeted and targeted analyses.

#### 2.7.1. Untargeted Mass Spectrometry

For Information-Dependent Acquisition (IDA) analysis, TL and CWL samples were resuspended in chloroform and methanol in 1:1 and 9:1 ratios, respectively. The samples were analyzed using an AB Sciex 4500 QTRAP MS/MS system equipped with a Sciex Turbo Spray ion source [Framingham, MA, USA] operating in the positive and negative ion modes. UHPLC was performed on an Agilent C18 column (100 mm × 2.1 mm, 1.8 μm) using a gradient elution over 41 min. Mobile phase A consisted of solution 2, which was prepared by mixing solution 1 [water/CAN (95/5, *v*/*v*) containing 5 mM ammonium acetate (AA)] with methanol in a 90:10 ratio (*v*/*v*). Mobile phase B consists of ACN:methanol:IPA in a ratio of 85:10:5 (*v*/*v*/*v*). The flow rate was set at 0.3 mL/min. The gradient for mobile phase B was 30% (0–15 min), increased to 50% (15–22 min), then to 85% (22–28 min), and returned to 30% (28–41 min). The source temperature was maintained at 500 °C, with the ion-spray voltage (IV) set to 5.50 kV, ion-source gases 1 and 2 both were 50 psi, the curtain gas was 30 psi, and the collision gas was set to medium. For each run, 2 µL of the lipid samples were injected. EMS survey scans were acquired over an *m*/*z* range of 200–2000, and precursor ions exceeding an intensity threshold of 500,000 cps were automatically selected for MS/MS fragmentation using IDA-EPI acquisition in linear ion trap mode. EPI spectra were recorded using rolling collision energy (40 ± 20 eV). The acquired fragmentation data were further processed for lipid annotation using the MS-LAMP workflow together with characteristic EPI fragmentation patterns [11].

#### 2.7.2. Targeted Mass Spectrometry

Targeted lipid profiling was conducted using the same resuspended samples on the same UHPLC-MS/MS system operated in multiple reaction monitoring (MRM) mode. Protonated precursor-to-product ion transitions were optimized for individual GL and GPL lipid species. Quantification was performed using SPLASH™ LIPIDOMIX™ as an internal standard alongside natural standards: TAG, DAG from GL lipid category, and PC, LPC, PE, PI, and PS from GPL lipid category. Chromatographic separation was achieved on a Kinetex^®^ C18 column [Torrance, CA, USA] (100 Å, 50 × 2.1 mm, 1.7 μm) using a binary gradient (two-solvent system) comprising Buffer A (water, 0.5% formic acid, 10 mM AA) and Buffer B (methanol, 0.5% formic acid, 10 mM AA) at a constant flow rate of 0.3 mL/min. A 30 min run was executed for GLs and 20 min for GPLs, targeting 125 GL lipid species and 71 GPL lipid species. For GL and GPL analyses, the gradient for mobile phase B was 80% (0–6 min) followed by an increase to 100%, maintained from 6 to 25 min for GL and 6–15 min for GPL, and then returned to 80% (25–30 min for GL; 15–20 min for GPL). The predefined Q1/Q3 transitions were adopted based on previously reported lipidomics studies [12]. The instrumental parameters were optimized in the positive ion mode, with a source temperature of 500 °C, ion-spray voltage of 4.5 kV, ion source gases 1 and 2 at 50 psi, curtain gas at 35 psi, and medium collision gas. These parameters were uniformly applied across targeted lipids. The autosampler and column oven were maintained at 10 °C and 60 °C, respectively. A 10 µL volume of each sample was injected, and data were acquired over the *m*/*z* 200–1000 range using Analyst software version 1.6.3 in wiff format.

#### 2.7.3. Quality Control Measurements for Mass Spectrometry

Quality control was conducted using natural standards of DAG, TG, PC, LPC, PE, PI, and PS, alongside a pooled sample matrix. All standards and test samples produced well-resolved, distinct chromatographic peaks, allowing for the accurate and absolute quantification of the targeted GLs and GPLs. In addition, the method was validated by determining the limit of detection (LOD) and limit of quantification (LOQ), which were assessed through serial dilutions of internal and natural standards (0.98–1000 ng/mL). Regression analysis was performed using Microsoft Excel (MS-Excel) to confirm the analytical performance of the developed UPLC-MS/MS method. Consistent results across biological and technical replicates supported the reproducibility of the analytical workflow.

### 2.8. High-Throughput Data Analysis

The acquired IDA data were processed and sorted in MS-Excel by selecting shared masses across triplicates with a minimum intensity threshold of 10,000 (E4) for further interpretation. MTB lipid species were identified using a mass spectrometry-based lipid(ome) analyzer and molecular platform (MS-LAMP) software-Version 1.0 with the MTB LipidDB for systematic lipid classification, as previously described [13]. Lipid annotation was based on accurate mass matching of [M^+^H]^+^ and [M^−^H]^−^ ions within a defined mass tolerance window (±0.5 *m*/*z*), consistent with previously reported approaches [9,14]. MS/MS fragmentation patterns were considered, where available, to support lipid assignments. The matched lipid masses from both ionization modes were combined for subsequent analysis.

For quantification, the MRM data were processed using MultiQuant v3.0 (Sciex) for peak smoothing and background subtraction. Calibration curves for TAG, DAG, PC, LPC, PE, PI, and PS were generated using internal and natural standards with matrix subtraction, achieving a correlation coefficient (R^2^) of 0.99. These curves enabled the absolute quantification of lipid species across clinical isolates.

### 2.9. Multivariate Statistical Analysis

Multivariate analysis, including principal component analysis (PCA), partial least squares discriminant analysis (PLS-DA), hierarchical clustering, data validation, and biomarker analysis, was performed using the web-based tool MetaboAnalyst 6.0 [10]. Additionally, shared and unique lipid species across all isolates were identified using Venny 2.1 software. Quantitative visualizations, i.e., doughnut charts and histograms for GL and GPL lipid species, were generated in MS-Excel.

## 3. Results

### 3.1. Merged Untargeted Lipidome Analysis Reveals Partial Phenotype Separations

To assess lipidomic variations among DS, DR, MDR, and PXDR MTB clinical isolates, an integrated analysis was conducted on merged untargeted lipidomic datasets derived from TL and CWL extracts. The radar plot (Figure S1A) showed comparable numbers of queried *m*/*z* features across phenotypes, but a consistently lower count of identified MTB-specific *m*/*z* values, indicating a selective lipid signature. Multivariate analysis (Figure S1B) revealed resistance-associated lipidomic shifting among MTB phenotypes. PCA (top panel) showed partial group separation, with PC1 (58%) and PC2 (15.6%) accounting for 73.6% of total variance. PXDR isolates displayed broader dispersion, whereas MDR strains showed emerging clustering. The PCA biplot of the first five PCs (cumulative variance ~97.9%) confirmed partial segregation. PLS-DA (bottom panel) further improved group resolution, with components 1 (52.3%) and 2 (20.6%) explaining 72.9% of the variance. The ANOVA plot (Figure S1C) identified polyketides (PKs) as the most discriminatory lipid class, followed by moderate shifts in FAs, GPLs, and prenol lipids (PRs), while GLs and saccharolipids (SLs) showed minimal variation. The Venn diagram (Figure S1D) demonstrated that 470 lipid species (38.1%) were conserved across all phenotypes, while DS isolates exhibited the highest number of unique lipids (*n* = 95), potentially representing drug-sensitive metabolic traits. DR and MDR isolates shared 59 unique lipids each, whereas PXDR isolates harbored 75 unique species, hinting at enhanced lipidomic diversification at higher resistance stages. Hierarchical clustering, dendrogram (Figure S1E), further supported these findings, revealing defined subclusters among resistant isolates and broader dispersion of DS isolates, indicative of transitional or evolving resistance states. Since merged datasets limited discrimination among clinical groups, TL and CWL lipidomes were reanalysed independently to uncover phenotype-specific lipidomic shifts associated with drug resistance.

### 3.2. CWL Profiling Exhibits PK-Driven Phenotype Separation, While TL Profiles Show Limited Variation

**Figure 1:**
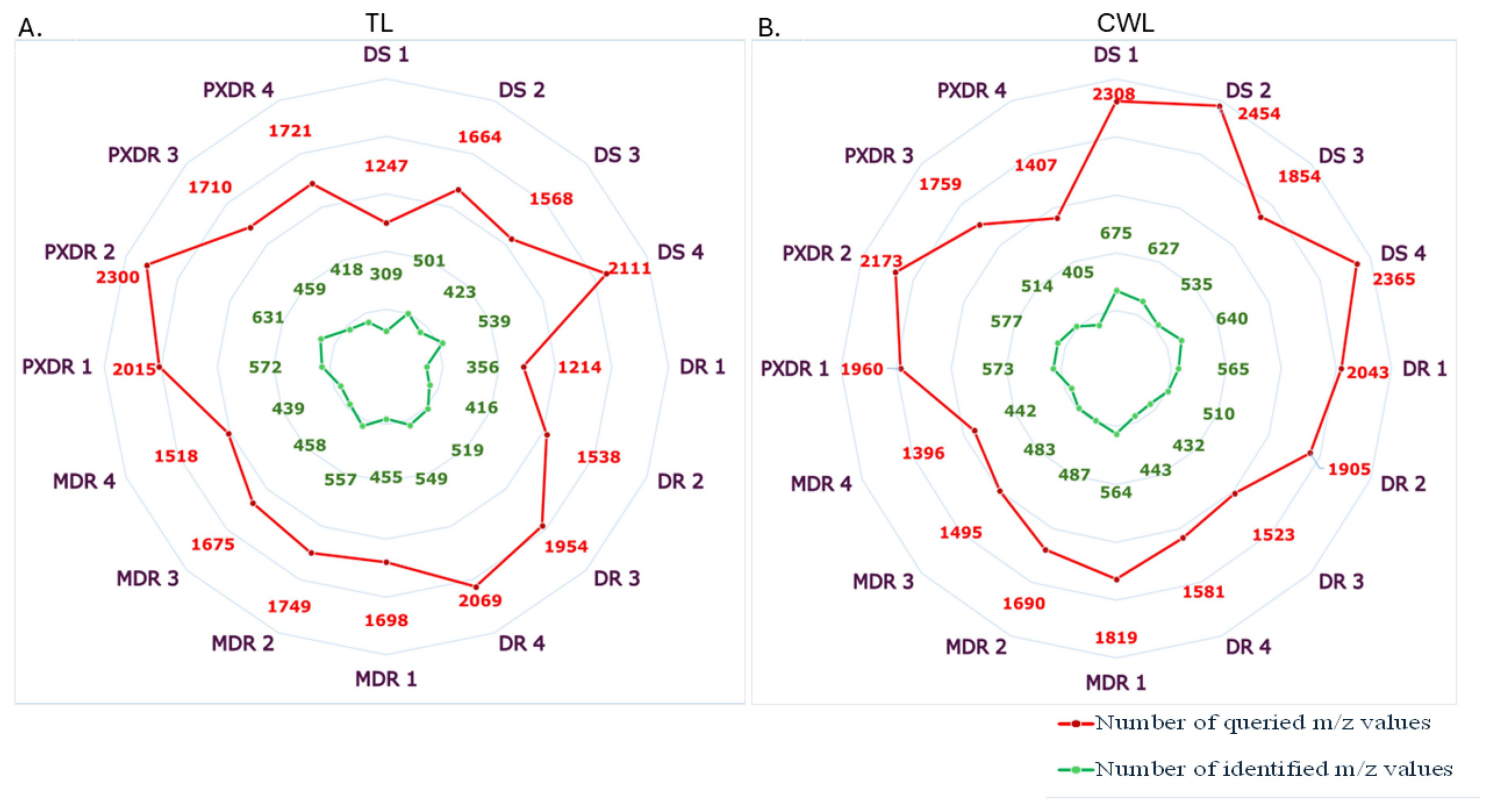
Radar plots showing the number of queried and identified *m*/*z* values in TL and CWL extracts of DS, DR, MDR, and PXDR MTB isolates. (**A**) TL extract and (**B**) CWL extract.

A wide array of lipids was identified within the *m*/*z* range of 300–1896 and 300–1882 in TL and CWL extracts, respectively (Table S1, Figure S2). In the TL extract, the number of quired *m*/*z* features ranged from 1214 to 2300, while identified lipids spanned 309 to 631 (Figure 1A), showing minimal variation among DS and resistant isolates. Comparatively, CWL extract (Figure 1B) exhibited higher quired *m*/*z* numbers (1396–2454) and a greater number of identified lipids (405–675), with DS isolates showing higher identified lipids compared to DR, MDR, and PXDR isolates.

**Figure 2:**
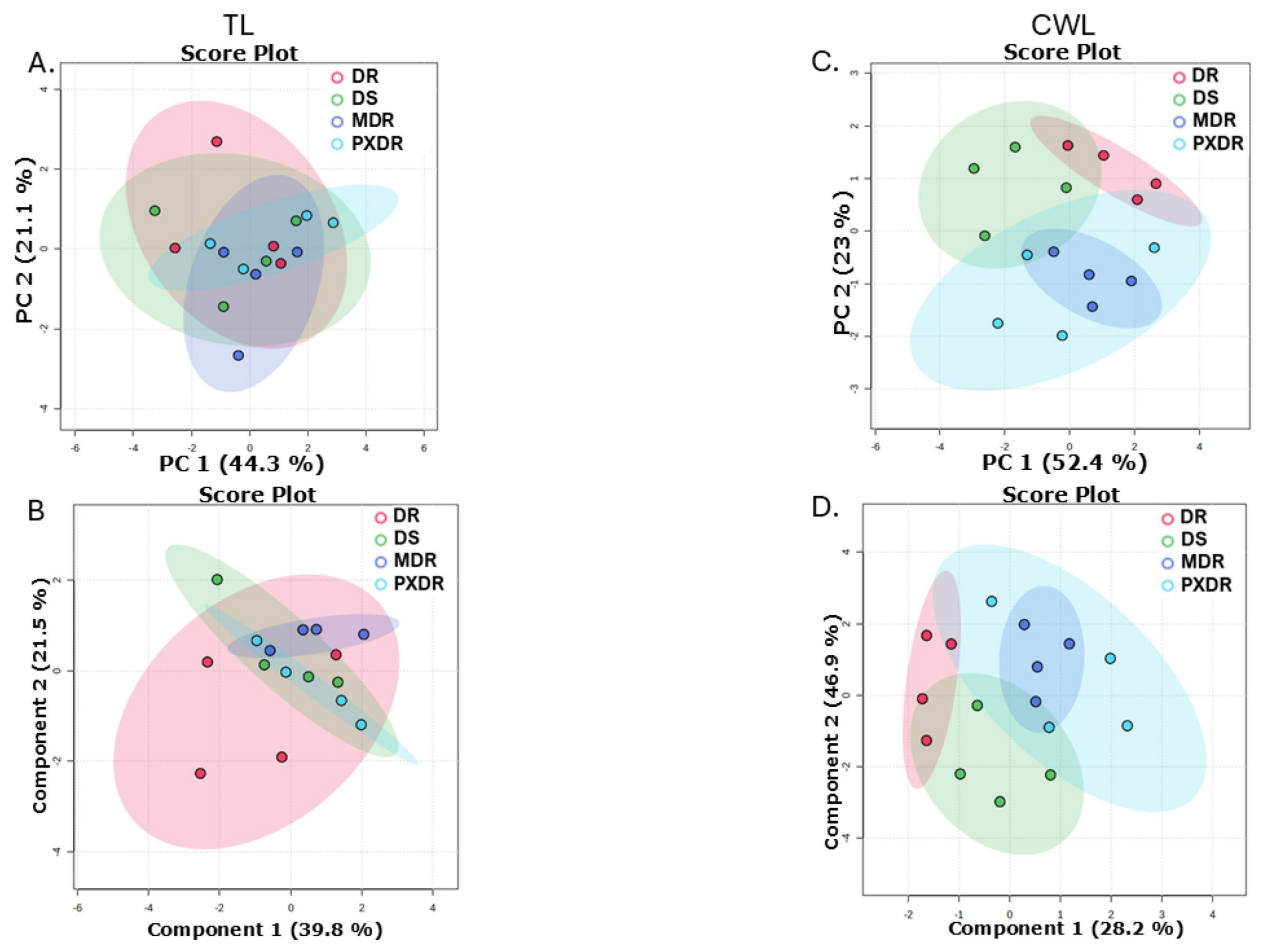
PCA (top panel) and PLS−DA (bottom panel) plots of TL and CWL lipidomic datasets across MTB resistance phenotypes. (**A**,**B**) TL extract and (**C**,**D**) CWL extract. Color coding for DR is red, DS is green, MDR is blue, and PXDR is cyan.

Multivariate statistical analyses were performed to assess lipidome patterns across MTB isolates. The PCA score plot for TL extracts showed limited distinction between MTB isolates (Figure 2A), with PC1 and PC2 accounting for 44.3% and 21.1% of the variance, respectively, (cumulative 65.4%). DR isolates formed a partially distinct cluster, while DS, MDR, and PXDR overlapped substantially. The first five PCs accounted for ~97.8% of the total variance, reaffirming weak discrimination among phenotypes. The PLS-DA plot (Figure 2B) explained 39.8% and 21.5% of the variance through components 1 and 2, respectively (total 61.3%), indicating modest separation. DS and PXDR isolates exhibited broader dispersion with substantial overlap, whereas DR isolates showed broader grouping, and MDR formed a tighter, more defined cluster. In contrast, CWL extract demonstrated pronounced phenotypic separation. PCA score plot (Figure 2C) exhibited a clear distinction between DS and resistant isolates, with PC1 and PC2 explaining 52.4% and 23% of the variance, respectively, totalling 75.4%; although MDR and PXDR clustered closely, they remained distinguishable. The PLS-DA plot (Figure 2D) further validated the discriminative strength of CWL profiles, accounting for 28.2% and 46.9% of variance in Components 1 and 2 (total 75.1%). It showed well-defined separation among DS, DR, MDR, and PXDR isolates, with DS and DR forming compact and distinct clusters, while MDR and PXDR exhibited broader dispersion, suggesting progressive lipidomic alterations associated with increasing drug resistance. In addition, lipid class-wise PCA was also performed for TL and CWL extracts to evaluate broader category-associated clustering trends across resistance phenotypes (Figure S3). Distinct clustering tendencies were observed across major lipid classes, although partial overlap among clinical isolates remained evident, indicating the biological variability among patient-derived samples.

**Figure 3:**
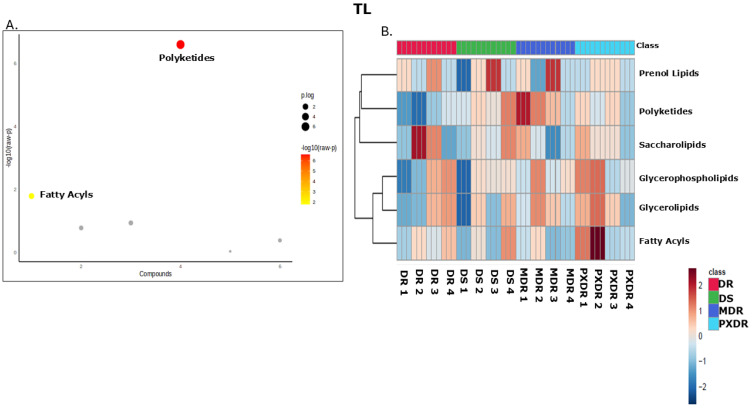

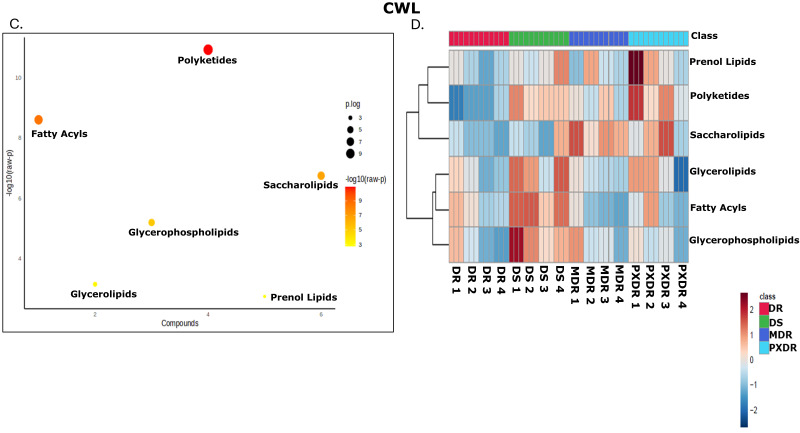
One−way ANOVA (Top panel) and heatmap (bottom panel) analysis of lipid categories in TL and CWL extracts. (**A**,**B**) In the TL dataset, the ANOVA bubble plot (top left) shows significant alteration in two lipid categories, i.e., FAs and PKs; the corresponding heatmap (bottom left) indicates minimal variation across DS, DR, MDR, and PXDR MTB isolates. (**C**,**D**) In the CWL dataset, the ANOVA bubble plot (top right) displays significant changes in all six lipid categories; the corresponding heatmap (bottom right) highlights distinct and well-defined differences in lipid profiles among the isolate groups.

One-way ANOVA and hierarchical clustering (heatmap) were employed to evaluate statistical significance and lipidomic divergence based on lipid categories across DS and resistant phenotypes (DR, MDR, PXDR). In TL extracts, the ANOVA bubble plot (Figure 3A) revealed that PK exhibited the highest statistical significance (−log_10_(*p*): 6.60), indicating marked differential abundance. FA showed moderate variation (−log_10_(*p*): 1.79), while GL, GPL, SL, and PL demonstrated low significance, suggesting relatively conserved distributions across phenotypes. This was consistent with the TL-based heatmap (Figure 3B), which showed minimal phenotypic separation, reflecting a broadly preserved lipidome within the TL compartment. Conversely, CWL-derived data showed markedly greater phenotypic resolution. The ANOVA bubble plot (Figure 3C) displayed marked divergence across all six lipid categories. PK again emerged as the most discriminative category (−log10(*p*): 10.92), followed by FA (8.60), SL (6.75), GPL (5.20), GL (3.15), and PR (2.75). The corresponding CWL heatmap (Figure 3D) reinforced this trend, where DS isolates exhibited higher abundance of FA, GL, and GPL classes, while resistant phenotypes showed elevated PK, PR, and SL levels. Notably, PK and PR levels peaked in PXDR isolates, highlighting progressive lipidomic remodeling aligned with escalating drug resistance, particularly within the cell wall lipidome.

**Figure 4:**
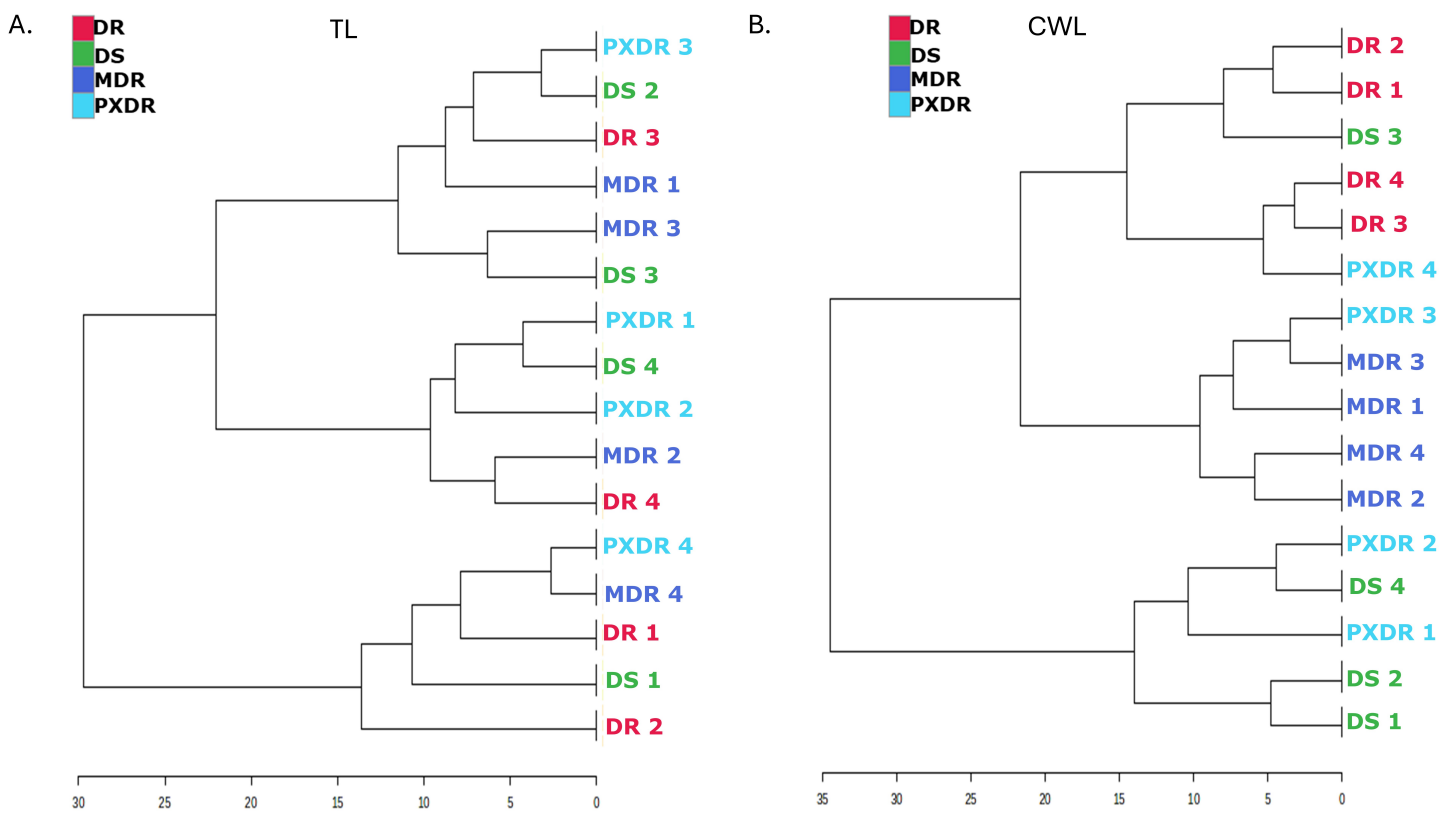
Hierarchical clustering dendrograms were generated from TL and CWL profiles of DS, DR, MDR, and PXDR isolates. (**A**) TL extracts show minimal separations among the four phenotypes, with substantial overlap across DS, DR, MDR, and PXDR isolates. (**B**) CWL extracts exhibit defined phenotype-specific clustering; drug-resistant isolates (DR, MDR, PXDR) form distinct and well-defined groups, and DS isolates partially cluster with PXDR and DR isolates.

Hierarchical clustering (dendrograms) provided further insight into phenotype-specific lipidomic structuring (Figure 4). TL-derived dendrogram (Figure 4A) revealed a high degree of intermixing among DS, DR, MDR, and PXDR isolates, reflecting internal heterogeneity and suggesting incomplete phenotypic separation within the TL extract. The frequent co-clustering of DS with resistant groups implies transitional or evolving lipidomic states potentially indicative of early-stage resistance. Comparatively, CWL-based dendrogram (Figure 4B) demonstrated markedly improved resolution, with resistant phenotypes forming more coherent and defined subclusters. DR isolates were tightly grouped, showing minimal overlap with DS, while MDR and PXDR isolates exhibited consistent intra-group clustering. Interestingly, a few DS isolates clustered near PXDR strains, possibly indicating early cell wall lipid remodeling in response to environmental pressures or subclinical resistance traits. These patterns underscore the superior discriminatory capacity of CWL profiles in delineating resistance-associated lipidomic shifts among MTB clinical isolates.

### 3.3. TL Extracts Show Neutral Lipid, Mycolate and PIM Shift, Whereas PK, PR and SL Elevations in CWL Extracts

**Figure 5:**
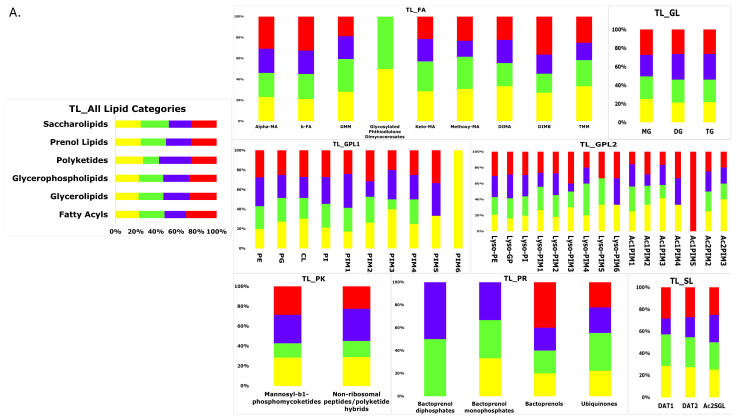

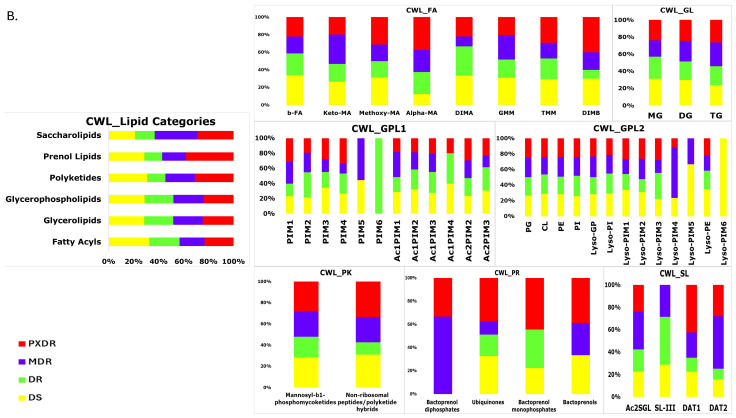
Relative abundance of lipid categories and their subclasses in TL and CWL extracts across DS, DR, MDR, and PXDR MTB isolates. (**A**) TL and (**B**) CWL extracts show the distribution of lipid categories, viz. FA, GL, GPL (GPL1 & GPL2), PK, PR, and SL, and their subclasses. Stacked bar plots illustrate compositional differences in lipid profiles across drug-sensitive and drug-resistant isolates. DS in green), DR in red, MDR in blue, and PXDR in cyan color.

To expand this lipidomic profiling at higher resolution, relative abundance and compositional diversity of lipid categories and their subclasses were analyzed across TL and CWL extracts derived from all MTB isolates (Figure 5). In TL extracts (Figure 5A; Table S2A), among the FA category, branched-chain fatty acids (BFAs) progressively increased from DS to PXDR isolates. Glycosylated phthiodiolone dimycocerosates were enriched in DS and DR but diminished in MDR and were absent in PXDR. Mycolic acid (MA) subclasses also varied; keto and methoxy MAs were elevated in DS and DR, while alpha MA and DIMB were abundant in PXDR, reflecting stage-wise modification of lipid structures. In the GL category, TAG and diacylglycerols (DAG) showed a resistance-associated increase. GPLs such as phosphatidylethanolamine (PE), phosphatidylinositol (PI), and their lysophospho-derivatives (Lyso-PE, Lyso-PI, Lyso-GP) were elevated in resistant isolates, while cardiolipin (CL) predominated in DS. Notably, PIM6 was exclusive to DS, PIM5 was absent in DR, and Ac1PIM5 was uniquely detected in PXDR. In the PRs, Bactoprenol diphosphate occurred in DR and MDR, whereas Bactoprenol monophosphate was undetectable in PXDR. Total Bactoprenol levels showed a mild increase in PXDR. PK and SL subclasses remained relatively unchanged across TL profiles.

On the other hand, CWL extracts (Figure 5B, Table S2B) show distinct subclass-level shifts. BFAs were enriched in DS compared to resistant strains. Methoxy MAs were prominent in DS and PXDR, whereas alpha MAs were highest in PXDR and lowest in DS. Monoacylglycerol (MAG) and DAG levels were higher in DS, while TAGs were slightly higher in MDR and PXDR. Within the GPL category, PG, CL, PE, Lyso-GP, Lyso-PI, Lyso-PE, and Lyso-PIMs were consistently elevated in DS. PIM6 was detected only in DR; PIM5 and Lyso-PIM5 were shared between DS and PXDR. PK subclasses were comparable between DS and PXDR but reduced in DR and MDR. In PRs, Bactoprenol diphosphate was present in MDR and PXDR only. SL subclasses such as DAT1 were detected in MDR and PXDR, DAT2 was exclusive to PXDR, and SLIII was absent in PXDR. These subclass-specific shifts potentially linked to resistance mechanisms and membrane adaptation.

### 3.4. Resistance-Specific Changes in Mycolates, TAGs, and PIMs with a Conserved Core Lipidome Across Different MTB Phenotypes

To dissect lipidomic overlaps and divergences across MTB phenotypes and extract types, three levels of Venn diagram analysis were conducted: intra-isolate extract comparison, inter-phenotype comparison, and comprehensive phenotype-wide comparison.

**Figure 6:**
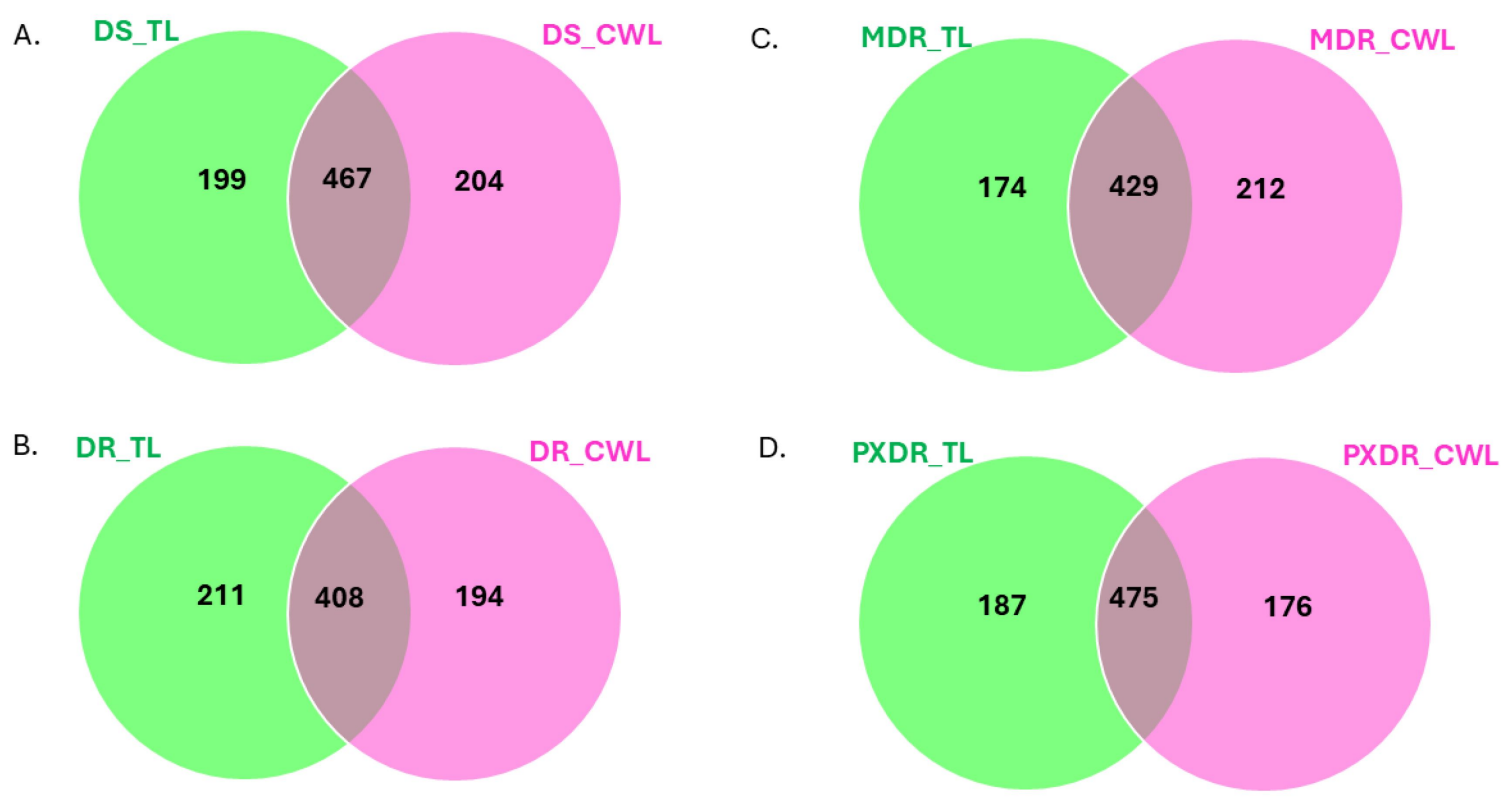
Venn diagrams showing intra-isolate extract comparison in TL and CWL extracts to identify unique and overlapped lipid species in DS, DR, MDR, and PXDR isolates. (**A**) Extract-wise comparison between DS isolates; (**B**) comparison between DR isolates; (**C**) comparison between MDR isolates; (**D**) comparison between PXDR isolates.

In intra-isolate extract comparison (Figure 6), DS isolates exhibited 467 overlapping lipids between TL and CWL, with 199 and 204 lipids unique to TL and CWL, respectively (Figure 6A). DR isolates shared 408 lipids between compartments, with 211 TL-exclusive and 194 CWL-exclusive lipids (Figure 6B). Similarly, MDR isolates exhibited 429 overlapping lipids to both extracts, and 174 and 212 lipids uniquely found in TL and CWL, respectively (Figure 6C). PXDR isolates showed the highest overlap (475 shared lipids), with 187 and 176 unique lipids in TL and CWL (Figure 6D). The corresponding lipid identities and molecular formulas are listed in Supplementary Material S1.

**Figure 7:**
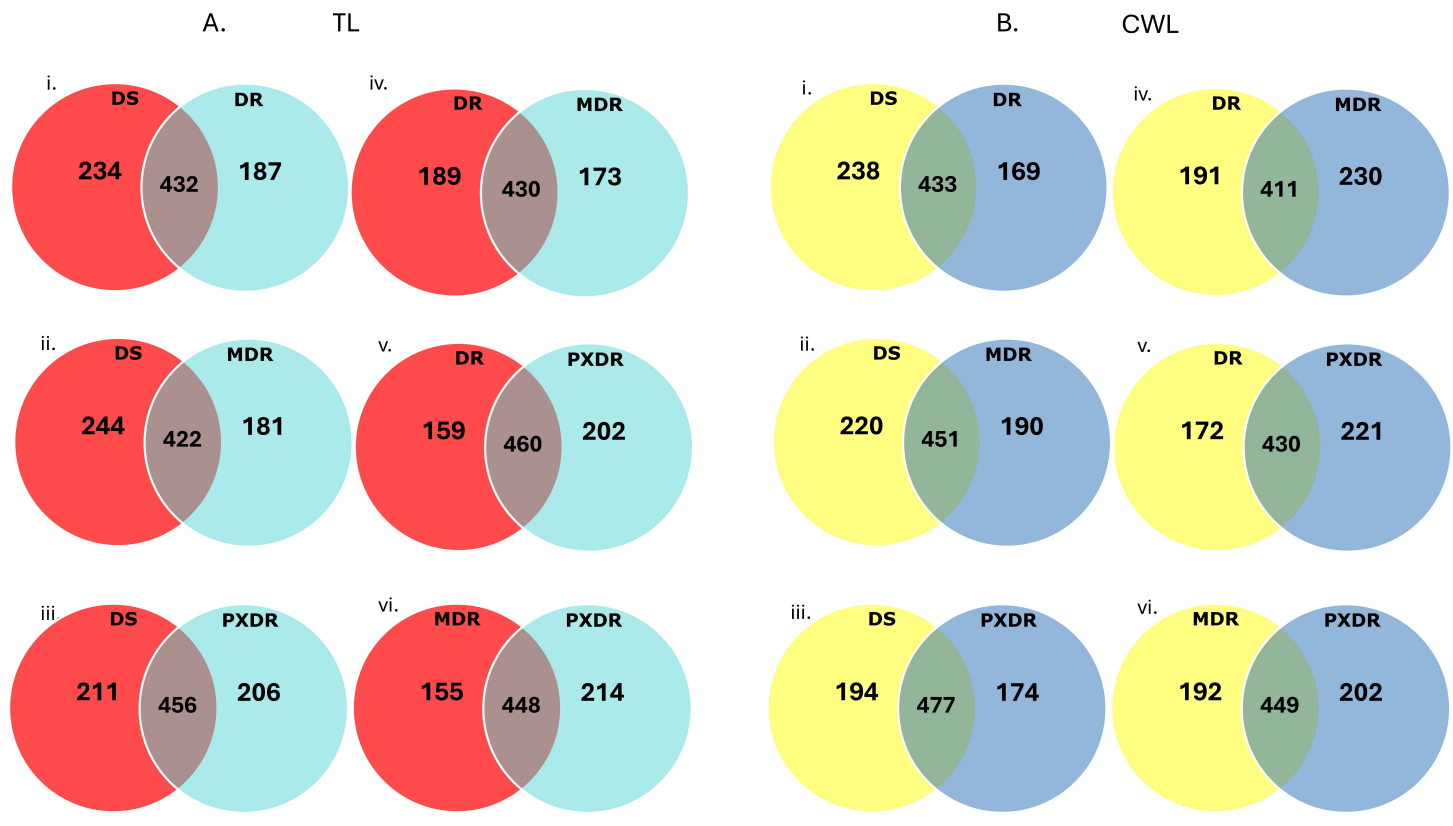
Venn diagrams showing inter-phenotype comparison of TL and CWL profiles between isolate groups. (**A**) TL extract—(i) DS vs. DR; (ii) DS vs. MDR; (iii) DS vs. PXDR; (iv) DR vs. MDR; (v) DR vs. PXDR; (vi) MDR vs. PXDR; (**B**) CWL extract—(i) DS vs. DR; (ii) DS vs. MDR; (iii) DS vs. PXDR; (iv) DR vs. MDR; (v) DR vs. PXDR; (vi) MDR vs. PXDR.

Inter-phenotype pairwise comparisons between phenotypes refined this lipid distribution (Figure 7). TL fractions showed 432 to 468 shared lipids between isolate pairs, with 155–244 lipids being exclusive per group (Figure 7A). CWL data comparisons followed similar trends, with 411–477 shared and 169–238 unique lipids (Figure 7B). All identified lipid species in Figure 7, along with their molecular formulas, are provided in the Supplementary Material S2.

**Figure 8:**
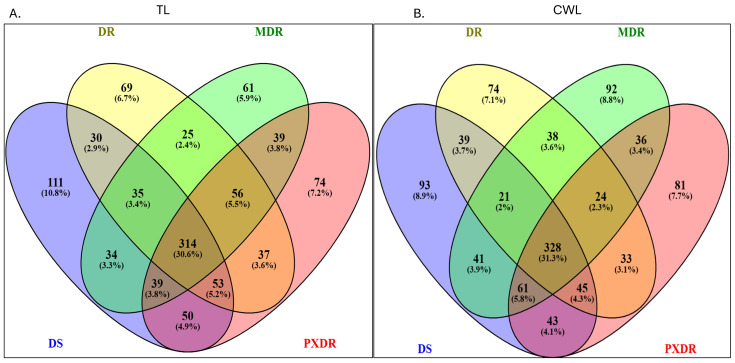
Venn diagrams representing a comprehensive phenotype-wide profiling of lipid species among DS, DR, MDR, and PXDR MTB isolates in TL and CWL extracts. (**A**) TL extract data and (**B**) CWL extract data.

Expanding this to a comprehensive phenotype-wide comparison (Figure 8), TL extracts showed DS, DR, MDR, and PXDR isolates uniquely contributed 111, 69, 61, and 74 lipid species, respectively (Figure 8A). Correspondingly, CWL fractions exhibited 93, 74, 92, and 81 exclusive lipids in DS, DR, MDR, and PXDR, respectively (Figure 8B). A conserved set of 314 (TL) and 328 (CWL) lipid species was shared among all phenotypes, while inter-group overlaps varied, indicating both conserved and resistance-associated molecular heterogeneity. Supplementary Material S3 provides the list of lipid molecules for Figure 8.

In this comparison, α-MAs were most abundant, followed by methoxy- and keto-MAs. α-MA (C89) was exclusive to PXDR, keto-MAs (C82, C93) to MDR, and methoxy-MA (C85) to DR. PGL-Tb (C-106) was exclusive to DS, while C-107 was shared by DS, DR, and PXDR, indicating its broader role in persistence across resistance phenotypes. PDIM lipid variants (DIM-B) declined with resistance, yet PXDR isolates showed selective retention of DIM-B (C102, C95, C97), possibly as an adaptive response to drug pressure. Specific glycosylated phthiodiolone dimycocerosate variants (e.g., C107, C110, C115 in DS; C103, C106 in DR) were distributed in a resistance-dependent manner. In contrast, core lipid classes such as mycocerosic, mycolipenic, and phthioceranic acids remained conserved, reflecting their fundamental role in MTB biology. In GL lipids, TAG (75:2) was specific to DS, TAG (76:0) to MDR, and TAGs (80:0, 85:0) exclusive to PXDR. In GPLs, a progressive decline in PIMs with increasing mannose residues was observed, and Ac2PIM4 was exclusive to PXDR.

Among the SLs, SL-III abundance also decreased in resistant strains. In contrast, diacyltrehaloses (DATs) remained conserved across isolates. However, DAT 1 (C56) was absent in DR and MDR but present in DS and PXDR isolates, while DAT 2 (C57) appeared exclusively in resistant strains. Altogether, these results highlight the coexistence of a stable core lipidome with phenotype- and compartment-specific variations. The observed increase in lipid diversity with higher resistance levels likely reflects adaptive physiological responses to escalating drug pressure.

### 3.5. Targeted Lipidomics Exhibits Higher GL Concentrations in MDR and PXDR, While GPLs Show Phenotype-Specific Distribution

**Figure 9:**
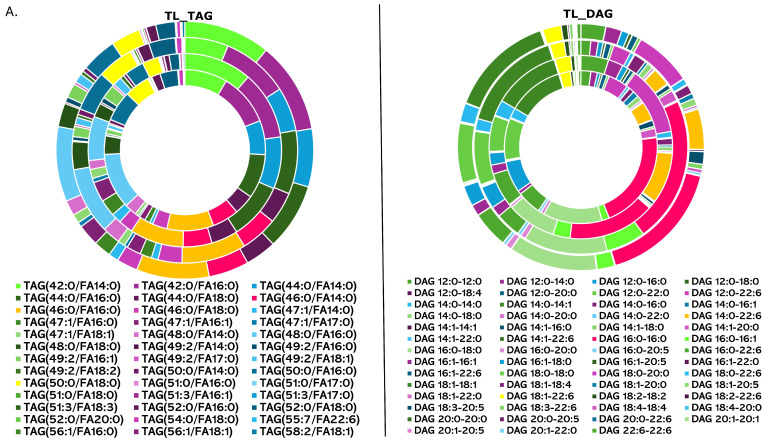

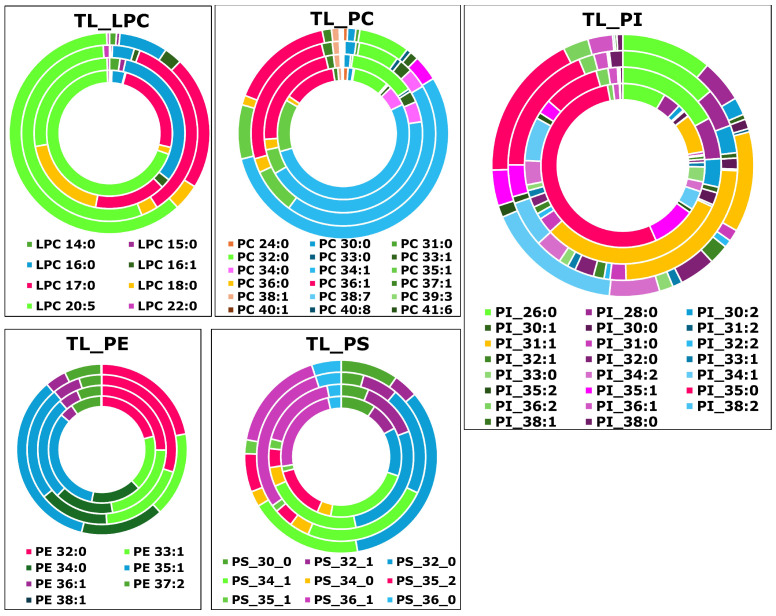

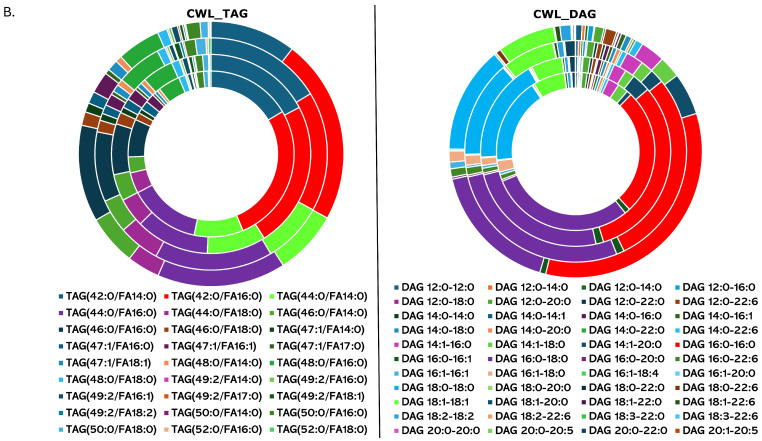

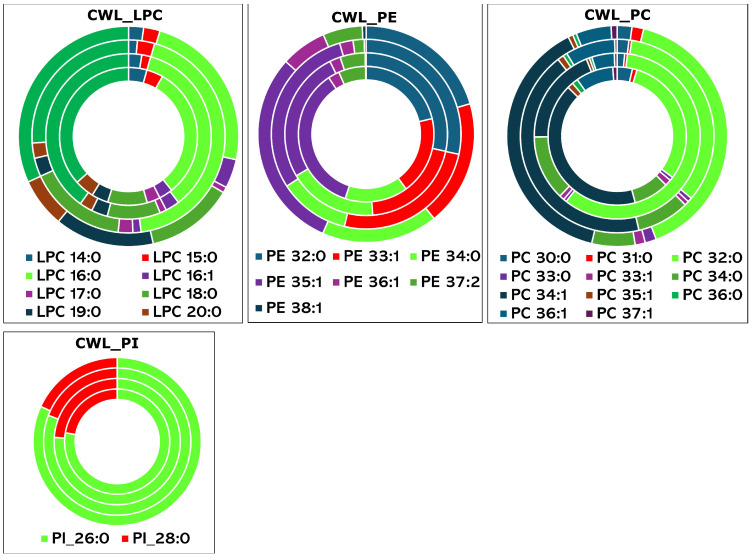
Sunburst plots illustrate the compositional distribution of quantified GL (TAG & DAG) and GPL (PC, PE, LPC, PI & PS), progressing from DS (center) to DR, MDR, and PXDR (outermost ring). (**A**) In TL extract, (**B**) in CWL extract.

Based on prior untargeted data analysis highlighting enrichment of GLs and GPLs, a targeted MRM-based lipidomic approach was employed to quantify 196 lipid species across MTB isolates (Figure S4). These included 42 TAGs and 83 DAGs under the GL category and 18 PCs, 10 LPCs, seven PEs, 24 PIs, and 12 PSs from the GPL category. In TL extracts, 39 TAGs, 56 DAGs, 18 PCs, eight LPCs, seven PEs, 23 PIs, and nine PSs were quantified, whereas CWL extracts showed reduced lipid diversity, quantifying 27 TAGs, 44 DAGs, 11 PCs, nine LPCs, seven PEs, and only two PIs, notably PS species lacking entirely in CWL extracts. Quantitative values are summarized in Table S3, and the compositional distribution is visualized through sunburst plots in Figure 9, progressing from DS (center) to DR, MDR, and PXDR (outermost ring).

In TL extracts, resistant isolates exhibited the highest concentrations of most TAG and DAG species, particularly TAG (42:0/FA14:0), TAG (44:0/FA16:0), and DAG (18:1–18:1), though DS retained higher levels of TAG (50:0/FA16:0) and TAG (50:0/FA18:0). LPCs such as LPC (14:0, 15:0, 16:0, 16:1, 17:0 & 18:0) were enriched in DR, while LPC (16:1, 17:0, 22:0) predominated in MDR, and LPC (20:5) highest in DS. PE (32:0 and 35:1) were prevalent across all groups, particularly in DR and MDR, while PCs like PC (32:0, 34:1 & 35:1) were most abundant in DR and PC (35:1 and 24:0) were relatively elevated in DS. PI and PS subclasses showed minimal variation across phenotypes in TL extracts (Figure 9A; Table S3A).

In CWL extracts, MDR isolates exhibited the highest TAG levels, especially TAG (42:0/FA16:0), TAG (44:0/FA16:0), and TAG (48:0/FA16:0). DAGs such as DAG (16:0–16:0), DAG (18:0–18:0), and DAG (18:1–18:1) were most abundant in PXDR. LPC concentrations were increased with resistance, being lowest in DS. PE lipids such as PE (35:1, 32:0 and 34:0) were enriched in DS, while PCs (PC 32:0, 34:0 and 36:1) peaked in DR, followed by MDR and PXDR. Only two PI species, PI (26:0) and PI (28:0), were quantified in CWL, with upward trends with increasing resistance levels (Figure 9B; Table S3B). These findings demonstrate phenotype- and extract-specific shifts in GL and GPL lipid abundance, in association with advancing resistance phenotypes.

### 3.6. Early Resistance Dominated by GPL Alterations and Advanced Resistance Driven by GL Variations

Following quantification, statistical analysis was performed to identify significantly altered lipid species with a *p* ≤ 0.05 value across six pairwise comparisons: DS vs. DR, DS vs. MDR, DS vs. PXDR, DR vs. MDR, DR vs. PXDR, and MDR vs. PXDR.

**Figure 10:**
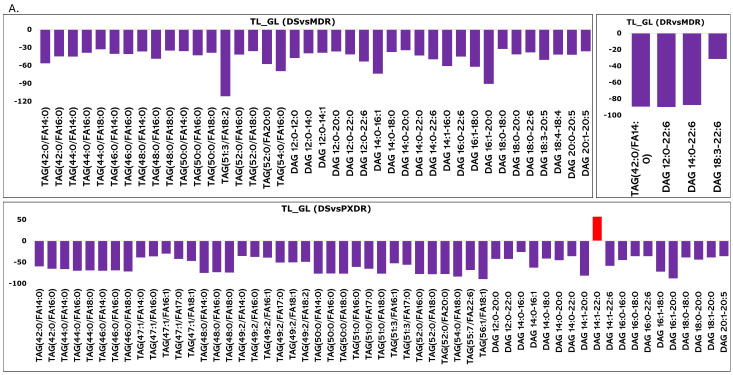

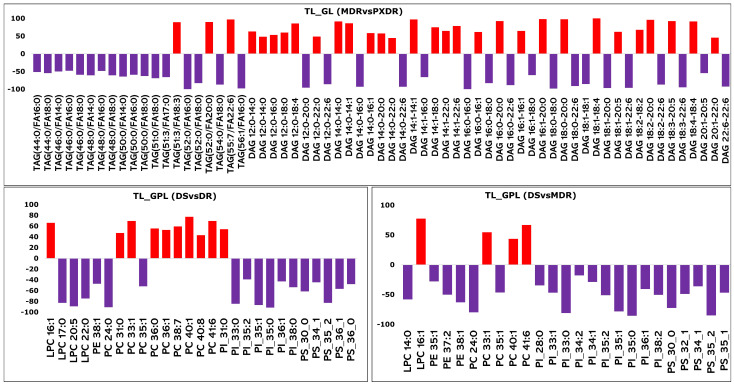

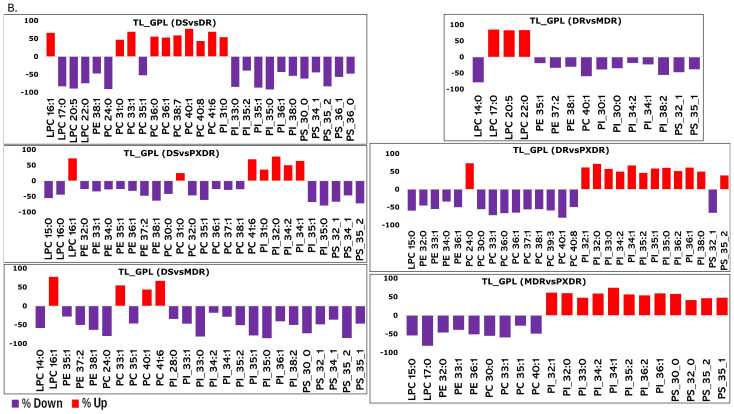
Vertical bar biplots showing differential regulation (up– or downregulation) of significant quantified lipids in TL extracts based on *p* ≤ 0.05 across six pairwise comparisons: DS vs. DR, DS vs. MDR, DS vs. PXDR, DR vs. MDR, DR vs. PXDR, and MDR vs. PXDR. Red bars indicate upregulation and purple bars indicate downregulation. (**A**) GL lipid species and (**B**) GPL lipid species.

In TL extracts (Figure 10A,B), no significant GLs were observed in DS vs. DR, though 27 GPLs were differentially expressed, including four LPCs, one PE, 10 PCs, seven PIs, and five PSs. In contrast, DS vs. MDR showed significant regulations in 40 GLs (17 TAGs, 23 DAGs) and 25 GPLs (two LPCs, three PEs, five PCs, 10 PIs, and five PSs), while DS vs. PXDR revealed the highest shift with 55 GLs (36 TAGs and 19 DAGs) and 27 GPLs (three LPCs, seven PEs, eight PCs, six PIs, and three PSs). Comparisons within resistant phenotypes, DR vs. MDR, showed four GLs (one TAG, three DAGs) and 15 GPLs (four LPCs, three PEs, one PC, five PIs, and two PSs) with significant changes. In DR vs. PXDR, no significant altered GLs were observed, but 28 GPLs were differentially regulated, comprising one LPC, four PEs, 10 PCs, 11 PIs, and two PSs. MDR and PXDR comparison showed the highest number of alterations with 65 significant GLs (20 TAGs, 45 DAGs) and 21 GPLs (two LPCs, three PEs, four PCs, eight PIs, and four PSs).

**Figure 11:**
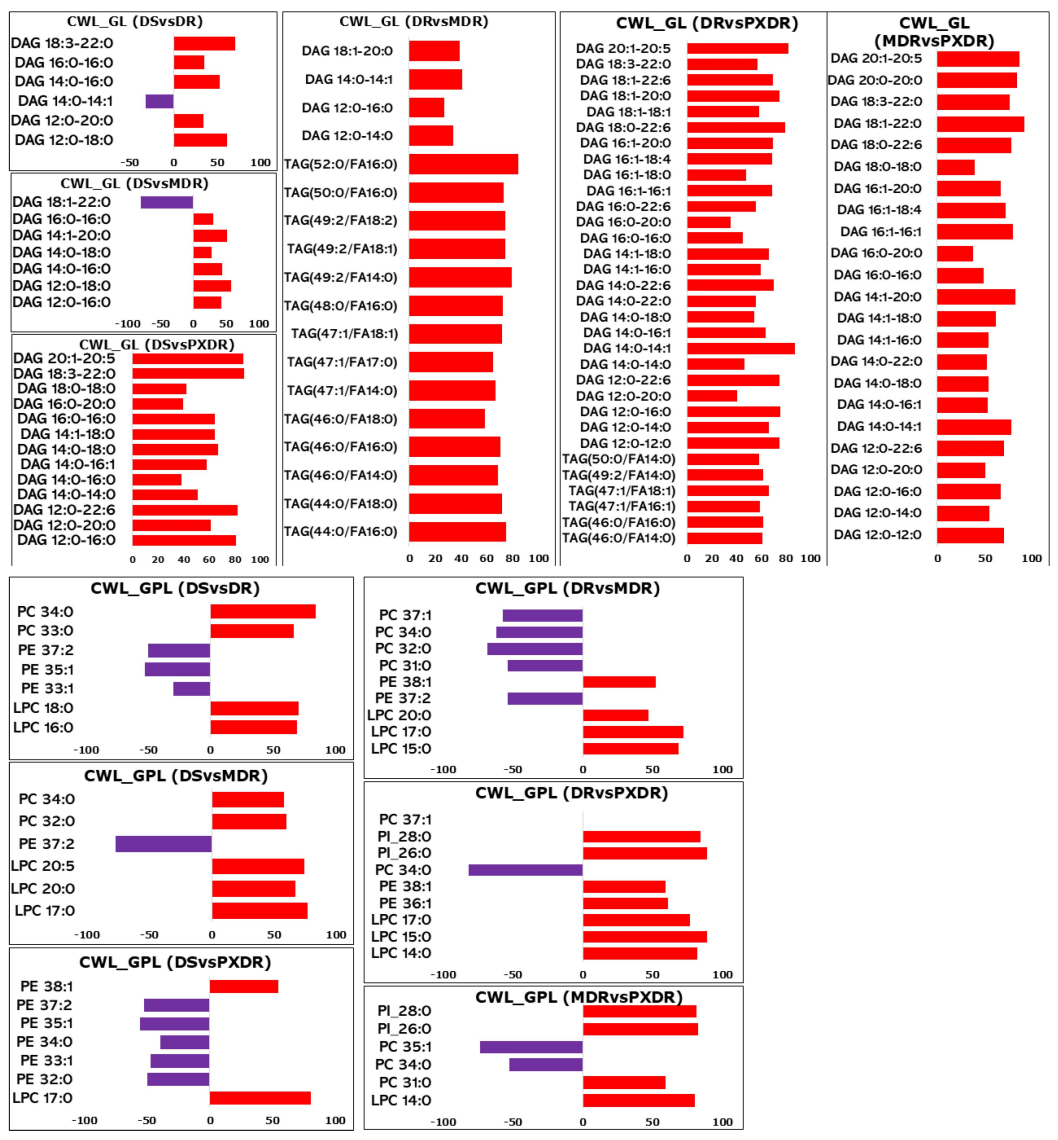
Horizontal bar biplots showing differential regulation (up– or downregulation) of significant quantified lipids in CWL extracts based on *p* ≤ 0.05 across six pairwise comparisons: DS vs. DR, DS vs. MDR, DS vs. PXDR, DR vs. MDR, DR vs. PXDR, and MDR vs. PXDR. Red bars indicate upregulation and purple bars indicate downregulation.

In CWL extracts (Figure 11), minimal changes were observed in drug-sensitive and resistant strains comparisons, while significant shifts emerged across resistant strains comparisons, indicating enhanced lipid perturbations with increasing resistance severity. DS vs. DR exhibited five significant GLs (all DAGs) and seven GPLs (two LPCs, three PEs, and two PCs). DS vs. MDR revealed seven GLs (all DAGs) and six GPLs (three LPCs, one PE, and two PCs), while DS vs. PXDR included 13 GLs (all DAGs) and seven GPLs (one LPC and six PEs) with significant regulation. The DR vs. MDR group had 18 GLs (14 TAGs and four DAGs) and nine GPLs (three LPCs, two PEs, and four PCs) with significant regulations. DR vs. PXDR comparison revealed 33 GLs (six TAGs and 27 DAGs) and eight GPLs (four LPCs, two PEs, one PC, and two PIs) as significantly regulated. Lastly, MDR vs. PXDR yielded 25 GLs (25 DAGs) and six GPLs (one LPC, three PCs, and two PIs) with significant differences. The complete list of statistically significant lipid species and corresponding *p*-values is provided in Table S4.

### 3.7. ROC-Based Analysis Identifies Compartment-Associated Lipid Signatures with Perfect Discriminatory Power (AUC = 1.0) Across MTB Phenotypes

Classical univariate Receiver Operating Characteristic (ROC) analysis was conducted on IDA datasets from both TL and CWL extracts to screen potential lipid biomarkers differentiating MTB phenotypes. The statistically significant lipids were selected based on *p* ≤ 0.05 value with Area Under the Curve (AUC) value of one and an optimal sensitivity-specificity balance determined using the Youden Index, and were considered to possess ideal discriminatory potential. Robustness of the analysis was ensured by computing 95% confidence intervals (CIs) through bootstrapping. A total of 18 lipid species (two FAs, nine GLs, six GPLs, and one PR) in TL (Figure S4A) and 34 lipids (six FAs, 10 GLs, 16 GPLs, one PK, and one PR) in CWL extracts (Figure S4B) were identified as significant discriminators across MTB phenotypes. A complete list of identified biosignatures is provided in Table 1.

## 4. Discussion

Despite ongoing advancements for STOP TB efforts, it continues to pose a challenge, further exacerbated by the emergence of drug-resistant MTB strains. The evolving spectrum of resistance from single-drug resistance to more complex forms like MDR, PXDR, and XDR-TB complicates therapy and raises concerns about treatment failure and transmission [15,16,17]. Given the pivotal role of the mycobacterial lipid envelope in pathogenicity, immune modulation, and drug tolerance, lipid-level insights are critical to understanding resistance-associated physiology [4,5,18]. The present study provides a systematic lipidomic comparison of drug-sensitive (DS) and resistant MTB isolates (DR, MDR, and PXDR), using both untargeted and targeted lipid profiling approaches. Each group included four biological replicates, each with three technical replicates for robust molecular-level profiling. In this study, OD600 (~1.0) was used only to standardize the growth phase of mycobacterial cultures prior to harvesting. As OD measurements may not accurately reflect equivalent biomass, lipid extraction was normalized by using cell lysate corresponding to 1 mg total protein for all isolates. This ensured comparable sample loading across all samples for both untargeted and targeted LC-MS analyses [10]. To capture a broad spectrum of lipid species, we employed two complementary extraction methods, i.e., TL extraction to recover structural and storage lipids, and CWL extraction to isolate cell wall-associated lipids linked to virulence and resistance [18,19]. This dual extraction strategy enabled a high-resolution view of compartment-associated lipid composition and its alterations across resistance phenotypes. While this strategy enables comparison between total and cell wall-associated lipid extracts, the fractions should be interpreted as operationally defined rather than strictly compartment-specific. The multivariate and statistical approaches applied in this study were intended to support the interpretation of complex lipidomic datasets by identifying consistent patterns across phenotypes. When integrated with validated lipid annotation, these approaches enable coherent interpretation of resistance-associated lipid remodeling, consistent with established lipidomics frameworks for biomarker discovery [7,20].

Qualitative profiling has been effective in the identification of strain-specific lipid species at high resolution. It also helps in uncovering lipid-based biomarkers that can distinguish drug-sensitive from drug-resistant MTB phenotypes [5,9,21]. A broad *m*/*z* range was scanned with both positive and negative ionization modes to maximize coverage of MTB-associated lipid species (Table S1). Initially, we analyzed merged TL and CWL datasets, which showed limited phenotypic distinctions (Figure S1). However, analysis of independent TL and CWL datasets showed defined phenotype-specific lipidomics alterations (Figure S2). CWL profiles demonstrated clear distinctions with resistance-associated shifting in all lipid categories (FA, GL, GPL, PK, PR, and SL) compared to TL profiling among MTB phenotypes (Figure 1, Figure 2 and Figure S3). Notably, ANOVA analysis (Figure 3) revealed significant alterations in PK species across both TL and CWL fractions, indicating perturbation of PK-associated lipid metabolism during resistance progression. Since PK-derived lipids are important components of the mycobacterial cell envelope and virulence machinery, their altered regulation may reflect adaptive cell envelope remodeling associated with reduced drug susceptibility [21,22,23,24,25]. However, membrane biophysical properties such as microfluidity were not directly assessed in the present study. Hierarchical clustering and dendrograms further supported these findings (Figure 4). These observations indicate that restructuring of CWL represents a key feature of resistance evolution and a potential marker of phenotypic divergence. Some previous studies have also reported that drug resistance in MTB is closely linked to the structure and function of the cell envelope, where many lipids influence permeability, virulence, immune interactions, and drug tolerance [5,6,9,22,23]. The observed variability in lipid profiles across certain groups, particularly in TL datasets, is likely reflective of underlying biological heterogeneity rather than analytical inconsistency. Since the study was performed on independent clinical isolates grouped by resistance phenotype, some degree of variation associated with host conditions and phenotypic differences is expected [3,26]. In MTB, resistance phenotypes, especially MDR and PXDR, arise through diverse genetic and metabolic adaptations rather than a single uniform pathway [4,27]. Therefore, the dispersion observed in multivariate analyses may represent biologically relevant heterogeneity associated with resistance evolution.

Subclass-specific analysis highlighted substantial and phenotype-specific mycobacterial lipid alterations across the drug resistance spectrum, from DS to PXDR MTB isolates (Figure 5A,B; Table S2). The progressive shift in BFAs from DS to PXDR indicates strengthened membrane packing and improved stress survival. Such changes are consistent with the role of methyl-branched lipids in modulating fluidity under antibiotic pressure [28]. MA subclasses also varied systematically with resistance severity. α-MAs were more abundant in PXDR isolates and aligned with previously reported adaptations in resistant strains where altered MA chain length and oxygenation enhance impermeability and antibiotic tolerance, particularly during rifampicin resistance acquisition [29,30]. These modifications are biologically significant because changes in MA subclass composition directly influence membrane fluidity, hydrophobicity, and drug penetration, which are central determinants of survival under prolonged drug pressure [23]. DS and early resistant isolates retained high levels of glycosylated phthiodiolone dimycocerosates, lipids known to aid macrophage entry and immune modulation [31,32]. These lipids are among the most energetically expensive virulence factors in MTB, requiring dedicated biosynthetic machinery [33]. Their loss in MDR and PXDR isolates suggests a strategic metabolic trade-off energetically costly virulence lipids in favor of envelope lipid configurations that better support persistence, structural resilience and drug tolerance under sustained antibiotic stress [3,23,27]. In contrast, PXDR isolates showed strong enrichment of DIMB, which may be advantageous when strains accumulate multiple resistance-conferring mutations that destabilize membrane homeostasis [34]. This reciprocal shift likely reflects a selective stabilization of the cell envelope in advanced resistance stages, over classical virulence functions.

GL metabolism also undergoes marked shifting during the transition from drug-sensitive to drug-resistant MTB. In TL extracts, DS strains exhibit higher levels of MAGs and DAGs, consistent with an intact and active TAG hydrolytic pathway that supports rapid fatty acid turnover and energy generation during exponential growth. Continuous TAG catabolism by mycobacterial lipases, such as Rv3097, leads to the transient accumulation of MAG and DAG intermediates, reflecting an energetically active metabolic state [9]. In contrast, MDR and PXDR strains display elevated TAG levels accompanied by reduced MAGs. It may be indicative of a dormancy-oriented metabolic reprogramming driven by increased TAG biosynthesis via triacylglycerol synthase (*tgs1*) and suppressed lipase activity, resulting in intracellular TAG accumulation within lipid droplets [35]. Moreover, resistance-associated increase in TAGs and DAGs in CWL extracts further supports their role as metabolic reserves during prolonged antibiotic stress [36]. Such lipid enrichment suggests modification of carbon flux toward energy-dense lipids, supporting metabolic survival and enhancing cell envelope hydrophobicity that limits drug penetration in drug-resistant MTB [23,37].

Similar to GPLs, the selective distribution of PIMs and their derivatives across resistance phenotypes indicates regulated reorganization of the PIM biosynthetic pathway during resistance evolution. Retention of PIM6 to DS and DR isolates suggests preservation of the canonical mannosylation cascade in these strains. PIM6 synthesis requires the PimF-mediated addition of the sixth mannose residue to PIM5, a step that also supports downstream flux toward lipomannan and lipoarabinomannan. Its presence reflects a lipid envelope optimized for immune engagement rather than drug stress adaptation. In contrast, disruption of this pathway in resistant strains, evidenced by the absence of PIM5 and emergence of Ac1PIM5 in PXDR, indicates adaptive remodeling of PIM processing. Acylated PIM intermediates have been shown to stabilize membrane architecture by increasing hydrophobicity and rigidity while bypassing energetically demanding mannosylation steps [38,39]. This pattern aligns with broader evidence that resistance evolution in MTB involves altered phosphoinositide flux to preserve membrane integrity under sustained drug stress [3,23].

PKs, PRs, and SLs are also crucial components of the MTB cell envelope, contributing to cell wall rigidity and stress adaptations [6,24]. In this study, the elevation of PKs and PRs in PXDR isolates, accompanied by increased SLs abundance in both MDR and PXDR strains, suggests targeted strengthening of the cell envelope as resistance advances [21,23]. Notably, these resistance-associated alterations were most apparent in the CWL extracts, supporting the previous reports that CWL profiling provides superior resolution of lipidomic shifts associated with drug resistance [5,9,23]. Overall, these findings support a model in which lipid alterations during resistance evolution reflect direct biochemical adaptation rather than incidental variation, enabling MTB to withstand prolonged drug pressure [29,34].

Multi-tiered Venn analysis showed that resistant MTB isolates retain a more selective lipid repertoire compared to sensitive MTB isolates (Figure 6, Figure 7 and Figure 8; Supplementary Material S1–S3). Dominance of longer-chain α-MAs (~C90) and TMM species in resistant phenotypes, consistent with an adaptive strategy of MTB that altered membrane permeability and limits drug entry as reported earlier [29,30,40]. Selective GL and GPL re-distribution further validates the resistance-associated shift in membrane architecture and metabolic reprogramming, with potential utility for biomarker development and therapeutic targeting [8,23]. In addition, the absence of Mbt-Fe (R = 17:0) in DR isolates suggests strain-specific adaptations to nutrient stress [41]. Earlier, some studies demonstrated that iron is essential for maintaining the metabolic stability and drug resistance in MTB. Limiting iron disrupted key cellular processes, weakening the bacterium’s defense mechanisms and enhancing its susceptibility to antibiotics [42,43,44]. Consistent with this, we observed a lower abundance of SL-III in resistant strains, supporting a shift toward retention of lipids more directly involved in survival under stress [26,45]. Increased levels of Ac2SGL with resistance further highlight the selective retention of lipids involved in immune modulation and drug tolerance, particularly rifampicin [27,46]. At the same time, retention of core lipid classes such as mycocerosic, mycolipenic, and phthioceranic acids, and DATs, occurs, reflecting their fundamental role in MTB biology and physiology [18,47].

Following quantitative MRM-based profiling of GL and GPL species, the untargeted findings were extended and validated, and revealed compartment-associated distinct lipid regulation patterns across resistance phenotypes (Figure 9; Table S3; Figure S4). In TL extracts (Figure 9A), enrichment TAGs and DAGs in MDR and PXDR are consistent with the strategy to respond linked to metabolic stress adaptation and energy conservation under antibiotic pressure [36,37]. Similarly, increased PEs and PCs concentrations in DR and MDR strains further reinforce restructuring of membrane phospholipids, while early resistance developed [28,48]. In CWL extracts (Figure 9B), resistance progression was marked by increased DAGs and LPCs, together with selective enrichment of specific PE and PC species, indicating compartment-associated membrane adaptation during resistance acquisition. The gradual increase in PIs with resistance further highlights the regulated phospholipid variations associated with envelope function [48]. In contrast, the consistent absence of PSs in CWL extracts across all phenotypes suggests a limited role in cell wall-associated resistance mechanisms.

Statistical comparisons of quantified lipids highlighted significant lipidomic shifts, especially between MDR and PXDR (Figure 10 and Figure 11; Table S4). In the TL extract (Figure 10A,B), significant alterations in GL and GPL at advanced resistance stages suggest that the development of extensive drug resistance is accompanied by substantial remodeling of the lipid profile rather than isolated lipid changes. In contrast, early resistant development might be associated with GPL species alterations, suggesting initial adaptation may preferentially target membrane phospholipid composition before more extensive lipid remodeling occurs.

The CWL extract analysis revealed a different pattern of lipid regulation. Here, the DR vs. PXDR comparison showed the highest number of significant changes (Figure 11). Interestingly, variable GLs regulation across different comparisons, either in TL extract or CWL extract, implies a role for neutral lipid metabolism in adapting to sustained drug pressure. The consistent involvement of PIs and PSs in many comparisons suggests that these phospholipids may be crucial in maintaining membrane properties conducive to drug resistance. This suggests that the progression from single-drug to pan-drug resistance involves significant modifications in cell wall-associated lipids, potentially contributing to enhanced drug efflux or reduced drug uptake [23,49]. Complementing this, biomarker analysis of untargeted datasets of TL and CWL extracts identifies lipids that could potentially differentiate between drug-sensitive and drug-resistant strains (Figure S5; Table 1). These lipid species reflect consistent classification across lipid categories, supported by MS-LAMP Mtb database-guided annotation and fragmentation evidence, and indicate coordinated lipid remodeling during resistance development. Notably, the panel of potential lipid markers shows consistent regulation across compartments, irrespective of their cellular localization. These findings underscore the importance of comprehensive lipid profiling in understanding the molecular basis of drug resistance in MTB and may contribute to the development of novel diagnostic tools for rapid identification of DR strains [21,23,50,51].

These findings collectively demonstrate that lipid shifting in MTB during the progression of drug resistance involves coordinated alterations in various lipid classes and differs between total cellular and cell wall compartments. Although in vitro culture without selective pressure may influence resistance-associated traits, the consistent lipid patterns observed across independent biological replicates indicate that the major phenotype-associated differences were retained under the conditions used. These observations further suggest that the lipid alterations reflect stable adaptations linked to resistance phenotypes rather than transient culture-induced variation. This comprehensive lipidomic profile provides valuable insights into potential lipid-based mechanisms of drug resistance. Further investigation into the functional roles of these differentially regulated lipids could provide valuable insights for developing new therapeutic strategies to combat drug-resistant tuberculosis.

Although this study was limited by the relatively small sample size of 16, the lipidomic findings were statistically validated and demonstrated strong discriminatory potential. The observed lipid alterations highlight phenotype-associated differences; however, these should be considered indicative trends rather than definitive conclusions. In addition, the analysis was performed at the phenotypic level (DS, DR, MDR, PXDR), and drug-specific associations were not explored. Since the clinical isolates were obtained from different patients, biological heterogeneity, including variations in strain background and clinical history, may also contribute to the observed lipidomic variability. Detailed treatment history of the isolates was also not available, which limits the correlation of lipid alterations with prior drug exposure. Although all isolates were phenotypically confirmed prior to lipid extraction and cultured under uniform conditions, prolonged in vitro growth in the absence of antibiotic pressure may have also influenced certain adaptive lipid responses. Further evaluation using larger, geographically diverse cohorts and functional assays will be required to confirm the diagnostic and mechanistic relevance of the proposed biomarkers. Additionally, integrating host–pathogen lipid interaction studies would further strengthen the translational applicability of these findings.

## 5. Conclusions

Comparative lipidomic analysis of drug-sensitive and resistant MTB isolates revealed distinct, phenotype-associated alterations across both TL and cell CWL fractions. Resistant phenotypes, particularly MDR and PXDR, exhibited enrichment of GLs, including increased TAG accumulation accompanied by reduced MAG levels in TL extracts, indicating a shift toward energy storage and metabolic adaptation. In parallel, CWL fractions showed alterations in GPLs, including changes in PIM species, along with modifications in FAs and MA profiles, reflecting remodeling of the cell envelope.

These changes progressed with resistance severity, where early resistance stages showed relatively subtle phospholipid variation, while advanced resistance was associated with broader reorganization of GL metabolism and CWL composition. Notably, lipid species derived from CWL fractions demonstrated higher discriminatory capacity between drug-sensitive and resistant phenotypes, supporting their potential as biomarkers.

Taken together, the data indicate coordinated but compartment-associated lipid alterations in lipid profiles across resistance phenotypes. These observations suggest that drug resistance in MTB is associated with systematic restructuring of both metabolic and structural lipid species. These findings provide a framework for further investigation of lipid-mediated mechanisms underlying resistance and highlight candidate lipid markers for future diagnostic and therapeutic exploration.

## Figures and Tables

**Table 1:** List of lipid species identified through biomarker analysis in TL and CWL extracts.

TL Extract	CWL Extract
**Fatty acyls (FAs)**	**Fatty acyls (FAs)**
Hydroxyphthioceranic acid (C46)	Mycocerosic acid (C27)
Phthioceranic acid (C43)	Mycocerosic acid (C28)
	Mycocerosic acid (C34)
**Glycerolipids (GLs)**	Mycocerosic acid (C35)
MG (RCO2H = 20:0)	Phthioceranic acid (C34)
DG (R1CO2H + R2CO2H = 31:2)	Phthioceranic acid (C40)
DG (R1CO2H + R2CO2H = 35:1)	
DG (R1CO2H + R2CO2H = 38:1)	**Glycerolipids (GLs)**
DG (R1CO2H + R2CO2H = 38:2)	DG (R1CO2H + R2CO2H = 31:0)
DG (R1CO2H + R2CO2H = 47:0)	DG (R1CO2H + R2CO2H = 32:0)
TG (R1CO2H + R2CO2H + R3CO2H = 46:0)	DG (R1CO2H + R2CO2H = 32:2)
TG (R1CO2H + R2CO2H + R3CO2H = 51:1)	DG (R1CO2H + R2CO2H = 34:2)
TG (R1CO2H + R2CO2H + R3CO2H = 53:2)	DG (R1CO2H + R2CO2H = 35:0)
	DG (R1CO2H + R2CO2H = 36:0)
**Glycerophospholipids (GPLs)**	DG (R1CO2H + R2CO2H = 38:0)
Lyso-PG (RCO2H = 18:1)	DG (R1CO2H + R2CO2H = 40:1)
Lyso-PI (RCO2H = 19:0)	DG (R1CO2H + R2CO2H = 41:1)
Lyso-PIM1 (RCO2H = 17:0)	TG (R1CO2H + R2CO2H + R3CO2H = 52:1)
PE (R1CO2H + R2CO2H = 37:0)	
PG (R1CO2H + R2CO2H = 30:1)	**Glycerophospholipids (GPLs)**
PG (R1CO2H + R2CO2H = 35:2)	Lyso-PG (RCO2H = 19:1)
	Lyso-PI (RCO2H = 18:0)
**Prenol Lipids (PR)**	Lyso-PI (RCO2H = 18:2)
DP-P	Lyso-PI (RCO2H = 19:2)
	Lyso-PIM1 (RCO2H = 18:1)
	Lyso-PE (R1CO2H = 18:1)
	PE (R1CO2H + R2CO2H = 30:0)
	PE (R1CO2H + R2CO2H = 31:1)
	PE (R1CO2H + R2CO2H = 32:2)
	PE (R1CO2H + R2CO2H = 34:2)
	PE (R1CO2H + R2CO2H = 36:1)
	PE (R1CO2H + R2CO2H = 37:2)
	PI (R1CO2H + R2CO2H = 33:0)
	PI (R1CO2H + R2CO2H = 33:1)
	PI (R1CO2H + R2CO2H = 35:2)
	PI (R1CO2H + R2CO2H = 36:1)
	PG (R1CO2H + R2CO2H = 42:1)
	PG (R1CO2H + R2CO2H = 45:0)

	**Polyketide (PK)**
	MPM (C30)

	**Prenol Lipids (PR)**
	MK-8 (H2)

## Data Availability

The original contributions presented in this study are included in the article/Supplementary Materials. Further inquiries can be directed to the corresponding authors.

## Supplementary Materials

The following supporting information can be downloaded at: https://www.mdpi.com/article/10.3390/life16060953/s1, Table S1: Overall and category-wise minimum and maximum queried and experimentally detected *m*/*z* values in TL & CWL extracts. Table S2: Distribution of experimentally detected and annotated lipid molecules across major lipid categories and their respective subclasses among DS, DR, MDR, and PXDR MTB clinical isolates. Table S3: Absolute concentrations for experimentally quantified lipid species from GL and GPL categories across DS, DR, MDR, and PXDR MTB isolates in TL and CWL extracts. Table S4: Percentage of differentially regulated GL (TAG and DAG) and GPL (PC, PE, LPC, PI, and PS) species derived from quantified lipids in TL and CWL extracts across DS, DR, MDR, and PXDR MTB isolates. Figure S1: Multivariate and statistical analysis of merged TL and CWL IDA data extracted from DS, DR, MDR, and PXDR MTB clinical isolates. Figure S2: IDA Chromatograms of isolates in Pos and Neg ionization mode. Figure S3: Lipid class-based PCA of TL and CWL extracts across DS, DR, MDR, and PXDR MTB isolates showing phenotype-associated clustering trends at the lipid category level. Figure S4: XIC Chromatograms of GL (TG & DG) and GPL (PC, LPC, PE, PI, & PS) categories acquired from targeted mass spectrometry. Figure S5: Box plots of potential lipid biomarkers identified in TL and CWL extracts of all referred MTB clinical isolates. Supplementary Material S1: List of exclusive and common lipid species experimentally detected and annotated in extraction-wise comparisons among DS, DR, MDR, and PXDR MTB isolates. Supplementary Material S2: List of exclusive and common lipid species experimentally detected and annotated in pair-wise comparisons among DS, DR, MDR, and PXDR MTB isolates in TL and CWL extracts. Supplementary Material S3: List of unique and shared lipid species experimentally detected and annotated across DS, DR, MDR, and PXDR MTB isolates in TL and CWL extracts.

## Abbreviations

**Abbreviation**	**Full Form**
ACN	Acetonitrile
AUC	Area Under the Curve
BFA	Branched Fatty Acids
BSL-3	Biosafety Level 3
CI	Confidence Interval
CL	Cardiolipin
CWL	Cell Wall Lipids
DAG	Diacylglycerol
DR	Drug-Resistant
DS	Drug-Sensitive
FA	Fatty Acyls
GL	Glycerolipids
GPL	Glycerophospholipids
IDA	Information-Dependent Acquisition
IPA	Isopropanol
KCl	Potassium Chloride
LC-MS	Liquid Chromatography-Mass Spectrometry
LOD	Limit of Detection
LOQ	Limit of Quantification
LPC	Lysophosphatidylcholine
MA	Mycolic Acids
MAG	Monoacylglycerol
MDR	Multidrug-Resistant
MRM	Multiple Reaction Monitoring
MS	Mass Spectrometry
MS-LAMP	Mass Spectrometry-Based Lipid(ome) Analyzer and Molecular Platform
MTB	*Mycobacterium tuberculosis*
OADC	Oleic Acid Albumin Dextrose Catalase
PC	Phosphatidylcholine
PCA	Principal Component Analysis
PE	Phosphatidylethanolamine
PG	Phosphatidylglycerol
PI	Phosphatidylinositol
PK	Polyketides
PLS-DA	Partial Least Squares Discriminant Analysis
PR	Prenol Lipids
PS	Phosphatidylserine
PXDR	Pre-extensively Drug-Resistant
QC	Quality Control
ROC	Receiver Operating Characteristic
SL	Saccharolipids
TAG	Triacylglycerol
TB	Tuberculosis
TL	Total Lipids
XDR	Extensively Drug-Resistant
