# Supplementary material for: Mapping of Lipidome Profile in Drug-Resistant Clinical Isolates of Mycobacterium tuberculosis Through Quali-Quantitative Liquid Chromatography-Mass Spectrometry Identifies Signature Lipids
Source: Life (Basel). 2026 Jun 5;16(6):953. doi: 10.3390/life16060953 (PMC13301348; doi:10.3390/life16060953)
Supplement: Supplementary file 1 [file life-16-00953-s001.zip › Supplementry tables and figure.pdf]

Table S1: Overall and category-wise minimum and maximum queried and experimentally detected m/z values in (a) TL & (b) CWL extracts.

a) m/z values for TL extract

	DS 1	DS 2	DS 3	DS 4	DR 1	DR 2	DR 3	DR 4	MDR 1	MDR 2	MDR 3	MDR 4	PXDR 1	PXDR 2	PXDR 3	PXDR 4
MINIMUM M/Z VALUES QUERIED	200.2	200.0	200.0	200.0	200.2	200.0	200.0	200.0	**200.0**	200.0	200.2	200.0	200.0	200.0	200.0	200.0
MAXIMUM M/Z VALUES QUERIED	1861.7	1897.0	1825.7	1769.6	1713.6	1894.2	1705.8	1727.2	1719.1	1786.9	1895.0	1806.0	1845.8	1891.4	1843.6	1712.3
MINIMUM M/Z VALUES IDENTIFIED	301.1	303.0	301.8	301.1	303.0	301.9	301.0	301.1	301.1	301.0	301.1	301.0	313.6	301.9	301.1	301.1
MAXIMUM M/Z VALUES IDENTIFIED	1767.4	1897.0	1872.1	1769.6	1743.8	1783.6	1437.5	1727.2	1719.1	1640.6	1895.0	1744.9	1637.4	1891.4	1843.6	1619.8
**Fatty Acyls**																
MINIMUM M/Z VALUES IDENTIFIED	367.3	369.2	367.3	381.2	381.2	367.2	367.4	367.3	367.3	369.2	370.0	367.3	369.2	369.2	369.2	369.2
MAXIMUM M/Z VALUES IDENTIFIED	1565.4	1811.6	1519.1	1769.6	1713.6	1783.6	1437.5	1727.2	1533.2	1499.3	1647.8	1727.6	1577.8	1772.0	1548.2	1619.8
**Glycerolipids**																
MINIMUM M/Z VALUES IDENTIFIED	301.8	303.1	301.8	301.8	301.9	301.9	301.0	301.1	301.1	301.0	301.1	301.0	303.1	301.9	301.1	301.1
MAXIMUM M/Z VALUES IDENTIFIED	1267.3	1297.2	1241.4	1199.3	1269.5	1299.0	1225.4	1226.6	1297.2	1339.0	1212.8	1239.6	1325.5	1252.7	1141.3	1297.2
**Glycerophospholipids**																
MINIMUM M/Z VALUES IDENTIFIED	450.2	450.4	464.3	450.5	452.3	452.2	450.2	452.5	450.1	451.0	452.9	452.2	452.2	450.2	450.2	453.0
MAXIMUM M/Z VALUES IDENTIFIED	1861.7	1897.0	1825.7	1769.6	1713.6	1759.6	1437.5	1727.2	1719.1	1786.9	1895.0	1744.9	1789.0	1891.4	1843.6	1619.8
**Polyketides**																
MINIMUM M/Z VALUES IDENTIFIED	681.6	708.1	681.7	680.2	708.4	682.1	694.2	695.6	680.0	679.6	694.0	829.0	681.7	707.6	682.0	694.1
MAXIMUM M/Z VALUES IDENTIFIED	923.5	910.2	925.7	923.8	925.6	871.1	925.6	908.3	925.6	882.1	895.9	923.6	898.3	910.1	882.0	925.7
**Prenol Lipids**																
MINIMUM M/Z VALUES IDENTIFIED	777.8	698.0	699.7	700.7	699.7	720.0	697.8	718.1	698.3	698.3	700.4	785.8	697.8	699.8	698.2	699.7
MAXIMUM M/Z VALUES IDENTIFIED	785.8	787.9	860.4	787.8	884.5	859.7	786.6	858.6	881.9	882.1	860.3	882.6	884.0	787.9	882.0	786.0
**Saccharolipids**																
MINIMUM M/Z VALUES IDENTIFIED	930.5	948.0	930.7	976.6	990.2	944.6	946.0	958.2	974.4	972.7	930.6	974.3	932.5	946.8	944.6	930.5
MAXIMUM M/Z VALUES IDENTIFIED	1295.3	1759.8	1196.3	1336.9	1168.8	1253.3	1364.6	1183.0	1362.6	1250.8	1335.4	1250.4	1252.8	1306.6	1154.4	1362.6

b) m/z values for CWL extract

	DS 1	DS 2	DS 3	DS 4	DR 1	DR 2	DR 3	DR 4	MDR 1	MDR 2	MDR 3	MDR 4	PXDR 1	PXDR 2	PXDR 3	PXDR 4
MINIMUM M/Z VALUES QUERIED	200.0	200.0	200.0	200.0	200.0	200.0	200.0	200.2	200.9	200.0	200.0	200.0	200.0	200.0	200.0	200.2
MAXIMUM M/Z VALUES QUERIED	1882.1	1717.1	1679.8	1771.8	1720.2	1771.7	1681.3	1781.6	1682.2	1875.5	1776.5	1577.5	1920.5	1787.0	1588.4	1783.3
MINIMUM M/Z VALUES IDENTIFIED	301.8	303.0	303.1	301.8	301.0	301.9	303.0	301.1	367.3	301.1	301.0	301.1	303.1	303.0	301.2	303.0
MAXIMUM M/Z VALUES IDENTIFIED	1882.1	1717.1	1673.2	1771.8	1690.0	1783.1	1681.3	1781.6	1661.3	1875.5	1765.7	1577.5	1763.8	1787.0	1553.3	1783.3
**Fatty Acyls**																
MINIMUM M/Z VALUES IDENTIFIED	369.4	367.2	369.2	369.2	369.2	369.4	369.2	369.4	367.3	367.2	383.2	384.1	370.1	369.2	367.2	369.2
MAXIMUM M/Z VALUES IDENTIFIED	1882.1	1691.8	1559.0	1771.8	1690.0	1771.7	1648.0	1537.8	1661.3	1631.6	1601.2	1577.5	1657.7	1577.6	1519.3	1783.3
**Glycerolipids**																
MINIMUM M/Z VALUES IDENTIFIED	301.8	303.0	303.0	301.8	301.0	301.9	303.0	301.1	301.2	301.1	301.0	301.1	303.1	303.0	301.2	303.0
MAXIMUM M/Z VALUES IDENTIFIED	1241.0	1285.3	1227.4	1339.4	1313.2	1252.7	1213.0	1311.6	1269.5	1139.0	1267.3	1182.7	1310.9	1243.6	1255.1	1297.3
**Glycerophospholipids**																
MINIMUM M/Z VALUES IDENTIFIED	450.2	450.5	450.2	450.5	450.5	452.3	452.3	450.2	452.9	451.0	450.2	450.4	452.2	450.4	452.3	452.6
MAXIMUM M/Z VALUES IDENTIFIED	1570.9	1717.1	1705.1	1765.3	1690.0	1771.7	1681.3	1781.6	1661.3	1875.5	1765.7	1577.5	1763.8	1787.0	1553.3	1783.3
**Polyketides**																
MINIMUM M/Z VALUES IDENTIFIED	681.5	681.8	681.5	681.7	707.5	680.0	679.6	696.4	695.6	707.8	682.0	679.4	679.4	680.2	679.7	696.4
MAXIMUM M/Z VALUES IDENTIFIED	895.9	923.8	912.4	893.5	857.5	926.4	855.4	921.5	923.5	925.9	925.8	923.6	924.0	898.3	923.6	911.8
**Prenol Lipids**																
MINIMUM M/Z VALUES IDENTIFIED	697.9	719.9	700.2	699.8	719.8	697.9	778.3	720.6	698.4	698.4	698.0	778.1	697.8	699.8	700.4	780.5
MAXIMUM M/Z VALUES IDENTIFIED	884.6	786.1	788.8	882.0	787.8	788.8	778.3	882.6	718.4	882.6	858.5	858.6	884.5	859.7	882.2	788.8
**Saccharolipids**																
MINIMUM M/Z VALUES IDENTIFIED	946.3	974.5	958.1	932.5	946.3	948.5	930.6	946.6	930.0	948.2	944.3	930.1	932.5	974.4	932.3	958.3
MAXIMUM M/Z VALUES IDENTIFIED	1293.1	1335.0	1673.2	1771.8	1661.3	1771.7	1208.6	1251.2	1661.3	1631.6	1306.7	1365.2	1266.6	1293.0	1320.6	1225.2

Table S2: Distribution of experimentally detected and annotated lipid molecules across major lipid categories and their respective subclasses among DS, DR, MDR, and PXDR MTB clinical isolates. (A) Lipid distribution in TL extract, (B) Lipid distribution in CWL extract

(A) TL Extract

**Lipid Categories**	**DS**	**DR**	**MDR**	**PXDR**
Fatty Acyls	84	90	77	108
Glycerolipids	167	178	189	195
Glycerophospholipids	167	172	185	194
Polyketides	12	7	14	11
Prenol Lipids	5	5	5	5
Saccharolipids	9	10	8	9

**Sub-Class of Fatty Acyls (FA)**	**DS**	**DR**	**MDR**	**PXDR**
Alpha Mycolic Acids (Alpha-MA)	3	3	3	4
Branched Fatty Acids (b-FA)	54	61	57	83
Glucose Monomycolates (GMM)	9	10	7	6
Glycosylated Phthiodiolone Dimycocerosates	1	1	0	0
Keto Mycolic Acids (Keto-MA)	4	4	3	3
Methoxy Mycolic Acids (Methoxy-MA)	4	4	2	3
Phthiocerol Dimycocerosates (DIMA)	3	2	2	2
Phthiodiolone Dimycocerosates (DIMB)	3	2	2	4
Trehalose Monomycolates (TMM)	4	3	2	3

**Sub-Class of Glycerolipids (GL)**	**DS**	**DR**	**MDR**	**PXDR**
Diacylglycerols (DG)	51	59	65	63
Monoacylglycerols (MG)	66	64	61	72
Triacylglycerols (TG)	50	55	63	60

**Sub-Class of Glycerophospholipids (GLP)**	**DS**	**DR**	**MDR**	**PXDR**
Diacylglycerolphosphoethanolamines (PE)	23	27	34	32
Diacylglycerophosphoglycerols (PG)	27	24	23	25
Diacylglycerophosphoglycerophosphodiradylglycerols (CL)	10	7	7	9
Diacylglycerophosphoinositols (PI)	14	16	18	18
Diacylglycerophosphoinositolmonomannosides (PIM1)	5	7	10	7
Diacylglycerophosphoinositoldimannosides (PIM2)	5	5	3	6
Diacylglycerophosphoinositoltrimannosides (PIM3)	4	1	3	2
Diacylglycerophosphoinositoltetramannosides (PIM4)	1	1	1	1
Diacylglycerophosphoinositolpentamannosides (PIM5)	1	0	1	1
Diacylglycerophosphoinositolhexamannosides (PIM6)	1	0	0	0
Monoacylglycerolphosphoethanolamines (Lyso-PE)	15	16	19	22

Monoacylglycerophosphoglycerols (Lyso-GP)	14	22	26	25
Monoacylglycerophosphoinositols (Lyso-PI)	8	10	11	12
Monoacylglycerophosphoinositolmonomannosides (Lyso-PIM1)	9	10	6	9
Monoacylglycerophosphoinositoldimannosides (Lyso-PIM2)	2	3	3	3
Monoacylglycerophosphoinositoltrimannosides (Lyso-PIM3)	3	2	1	4
Monoacylglycerophosphoinositoltetramannosides (Lyso-PIM4)	1	2	1	1
Monoacylglycerophosphoinositolpentamannosides (Lyso-PIM5)	1	1	0	1
Monoacylglycerophosphoinositolhexamannosides (Lyso-PIM6)	1	0	1	1
Monoacylated diacylglycerophosphoinositolmonomannosides (Ac1PIM1)	8	10	9	5
Monoacylated diacylglycerophosphoinositoldimannosides (Ac1PIM2)	7	5	3	6
Monoacylated diacylglycerophosphoinositoltrimannosides (Ac1PIM3)	5	2	3	2
Monoacylated diacylglycerophosphoinositoltetramannosides (Ac1PIM4)	1	0	1	1
Monoacylated diacylglycerophosphoinositolpentamannosides (Ac1PIM5)	0	0	0	1
Diacylated diacylglycerophosphoinositoldimannosides (Ac2PIM2)	2	2	2	2
Diacylated diacylglycerophosphoinositoltrimannosides (Ac2PIM3)	2	1	1	1

**Sub-Class of Polyketides (PK)**	**DS**	**DR**	**MDR**	**PXDR**
Mannosyl-b1-phosphomycoketides	4	2	4	4
Non-ribosomal peptides/polyketide hybrids	9	5	10	7

**Sub-Class of Prenol Lipids (PR)**	**DS**	**DR**	**MDR**	**PXDR**
Bactoprenol diphosphates	0	1	1	0
Bactoprenol monophosphates	1	1	1	0
Bactoprenols	1	1	1	2
Ubiquinones	2	3	2	2

**Sub-Class of Saccharolipids (SL)**	**DS**	**DR**	**MDR**	**PXDR**
2,3-di-O-acyltrehaloses (DAT1)	2	2	1	2

2,3-di-O-acyltrehaloses (DAT2)	3	3	2	3
Diacylated Sulfolipid (Ac2SGL)	4	4	4	4

(B) CWL Extract

Lipid Categories	DS	DR	MDR	PXDR
Fatty Acyls	137	102	84	98
Glycerolipids	219	180	181	191
Glycerophospholipids	234	189	199	193
Polyketides	17	8	13	17
Prenol Lipids	6	3	4	8
Saccharolipids	8	6	13	11

Sub-Class of Fatty Acyls (FA)	DS	DR	MDR	PXDR
Branched Fatty Acids	107	79	61	70
Keto Mycolic Acids (Keto-MA)	4	3	5	3
Methoxy Mycolic Acids (Methoxy-MA)	5	3	3	5
Alpha Mycolic Acids (Alpha-MA)	1	2	2	3
Phthiocerol Dimycocerosates (DIMA)	3	3	1	2
Glucose Monomycolates (GMM)	9	6	8	6
Trehalose Monomycolates (TMM)	5	4	3	5
Phthiodiolone Dimycocerosates (DIMB)	3	1	2	4

Sub-Class of Glycerolipids (GL)	DS	DR	MDR	PXDR
Monoacylglycerols (MG)	79	66	48	61
Diacylglycerols (DG)	84	62	68	69
Triacylglycerols (TG)	56	52	66	62

Sub-Class of Glycerophospholipids (GLP)	DS	DR	MDR	PXDR
Diacylglycerophosphoinositolmonomannosides (PIM1)	7	5	9	10
Diacylglycerophosphoinositoldimannosides (PIM2)	4	6	5	4
Diacylglycerophosphoinositoltrimannosides (PIM3)	5	3	3	4
Diacylglycerophosphoinositoltetramannosides (PIM4)	1	1	1	1
Diacylglycerophosphoinositolpentamannosides (PIM5)	1	0	1	0
Diacylglycerophosphoinositolhexamannosides (PIM6)	0	1	0	0
Monoacylated diacylglycerophosphoinositolmonomannosides (Ac1PIM1)	10	7	12	6
Monoacylated diacylglycerophosphoinositoldimannosides (Ac1PIM2)	6	5	4	4
Monoacylated diacylglycerophosphoinositoltrimannosides (Ac1PIM3)	2	2	2	2

Monoacylated diacylglycerophosphoinositoltetramannosides (Ac1PIM4)	1	1	0	1
Diacylated diacylglycerophosphoinositoldimannosides (Ac2PIM2)	2	2	2	3
Diacylated diacylglycerophosphoinositoltrimannosides (Ac2PIM3)	1	1	1	1
Diacylglycerophosphoglycerols (PG)	33	29	31	31
Diacylglycerophosphoglycerophosphodiradylglycerols (CL)	12	10	9	10
Diacylglycerolphosphoethanolamines (PE)	36	29	32	31
Diacylglycerophosphoinositols (PI)	19	20	18	18
Monoacylglycerophosphoglycerols (Lyso-GP)	28	22	26	23
Monoacylglycerophosphoinositols (Lyso-PI)	12	11	10	9
Monoacylglycerophosphoinositolmonomannosides (Lyso-PIM1)	11	7	6	9
Monoacylglycerophosphoinositoldimannosides (Lyso-PIM2)	5	3	4	4
Monoacylglycerophosphoinositoltrimannosides (Lyso-PIM3)	2	3	2	3
Monoacylglycerophosphoinositoltetramannosides (Lyso-PIM4)	1	0	3	1
Monoacylglycerophosphoinositolpentamannosides (Lyso-PIM5)	1	0	1	0
Monoacylglycerolphosphoethanolamines (Lyso-PE)	34	24	19	22
Monoacylglycerophosphoinositolhexamannosides (Lyso-PIM6)	1	0	0	0

**Sub-Class of Polyketides (PK)**	**DS**	**DR**	**MDR**	**PXDR**
Mannosyl-b1-phosphomycoketides	7	5	6	7
Non-ribosomal peptides/polyketide hybrids	10	4	8	11

**Sub-Class of Prenol Lipids (PR)**	**DS**	**DR**	**MDR**	**PXDR**
Bactoprenol diphosphates	0	0	2	1
Ubiquinones	4	2	1	4
Bactoprenol monophosphates	1	1	0	1
Bactoprenols	2	0	1	2

**Sub-Class of Saccharolipids (SL)**	**DS**	**DR**	**MDR**	**PXDR**
Diacylated Sulfolipid (Ac2SGL)	4	4	6	4
Sulfolipid III (SL-III)	1	1	1	0
2,3-di-O-acyltrehaloses (DAT1)	2	1	2	4
2,3-di-O-acyltrehaloses (DAT2)	1	1	4	2

Table S3: Absolute concentrations for experimentally quantified lipid species from GL and GPL categories across DS, DR, MDR, PXDR MTB isolates in TL and CWL extracts. (A) TL extract, (B) CWL extract

(A) TL Extract

Analyte (GL)	DS (ng/mL)	DR (ng/mL)	MDR (ng/mL)	PXDR (ng/mL)
TAG (42:0/FA14:0)	7.1732	14.8585	3.1179	2.8630
TAG (42:0/FA16:0)	8.7480	14.9762	4.8202	3.0242
TAG (44:0/FA14:0)	5.7157	11.3556	3.1361	1.9250
TAG (44:0/FA16:0)	7.4562	12.4959	4.5516	2.2125
TAG (44:0/FA18:0)	3.3880	5.8406	2.2650	1.0323
TAG (46:0/FA14:0)	4.4640	6.3077	2.6506	1.3348
TAG (46:0/FA16:0)	7.8334	11.7754	4.6231	2.4062
TAG (46:0/FA18:0)	2.4732	3.2073	1.7114	0.6968
TAG (47:1/FA14:0)	0.5431	2.1636	0.4824	0.3306
TAG (47:1/FA16:0)	0.9104	3.6724	0.8462	0.5796
TAG (47:1/FA16:1)	1.0292	4.3861	1.0085	0.7180
TAG (47:1/FA17:0)	0.2705	0.9377	0.2432	0.1561
TAG (47:1/FA18:1)	0.5706	2.0023	0.5169	0.3008
TAG (48:0/FA14:0)	2.0357	1.9490	1.2894	0.5055
TAG (48:0/FA16:0)	9.3702	9.6465	4.7901	2.4809
TAG (48:0/FA18:0)	3.0940	3.0874	2.0031	0.7838
TAG (49:2/FA14:0)	0.0805	0.3805	0.0774	0.0517
TAG (49:2/FA16:0)	0.2794	1.1292	0.2743	0.1744
TAG (49:2/FA16:1)	0.7905	3.1323	0.7420	0.4786
TAG (49:2/FA17:0)	0.1670	0.4713	0.1439	0.0815
TAG (49:2/FA18:1)	0.6126	1.9445	0.4976	0.3033
TAG (49:2/FA18:2)	0.2372	0.8603	0.2219	0.1202

Analyte (GPL)	DS (ng/mL)	DR (ng/mL)	MDR (ng/mL)	PXDR (ng/mL)
LPC 14:0	0.9775	1.9054	0.4080	0.8438
LPC 15:0	0.9375	1.0358	0.9136	0.4208
LPC 16:0	10.5344	27.2381	10.0399	5.8652
LPC 16:1	0.7062	1.7754	2.6973	2.1865
LPC 17:0	75.9482	12.9815	88.0848	16.2115
LPC 18:0	5.6485	16.2766	7.7135	3.2238
LPC 20:5	214.7060	23.1223	136.7955	46.2011
LPC 22:0	1.8693	0.4737	2.9018	0.3443
PE 32:0	1.4939	2.0056	2.0357	1.0982
PE 33:1	1.1996	1.7667	1.3004	0.7968
PE 34:0	1.0844	1.1907	1.0351	0.7859
PE 35:1	2.3542	2.1035	1.6979	1.7413
PE 36:1	0.2939	0.3977	0.4082	0.2000
PE 37:2	0.6667	0.4988	0.3313	0.3488
PE 38:1	0.0195	0.0103	0.0072	0.0071
PC 24:0	0.7760	0.0718	0.1567	0.2657
PC 30:0	0.8748	1.1456	1.1291	0.5081
PC 31:0	0.1883	0.3561	0.3485	0.2507
PC 32:0	5.9126	11.0888	4.0944	3.1459
PC 33:0	0.2899	0.4837	0.4738	0.2856
PC 33:1	0.5918	1.9388	1.3043	0.5336
PC 34:0	3.3420	3.4108	1.9966	1.5741

TAG (50:0/FA14:0)	0.6397	0.6698	0.4082	0.1465
TAG (50:0/FA16:0)	5.0795	4.2465	2.8914	1.1793
TAG (50:0/FA18:0)	4.2463	3.6298	2.5936	0.9717
TAG (51:0/FA16:0)	0.1578	0.2117	0.1140	0.0611
TAG (51:0/FA17:0)	0.1102	0.1105	0.0966	0.0382
TAG (51:0/FA18:0)	0.2499	0.2936	0.1828	0.0571
TAG (51:3/FA16:1)	0.1582	0.5335	0.1457	0.0748
TAG (51:3/FA17:0)	0.0161	0.0680	0.0206	0.0094
TAG (51:3/FA18:3)	0.0027	0.0072	0.0037	0.0024
TAG (52:0/FA16:0)	1.4751	1.1041	0.8590	0.3235
TAG (52:0/FA18:0)	2.9797	2.2033	1.9016	0.6409
TAG (52:0/FA20:0)	0.1573	0.1368	0.0664	0.0448
TAG (54:0/FA18:0)	0.7935	0.5041	0.4072	0.1267
TAG (55:7/FA22:6)	0.0050	0.0073	0.0039	0.0016
TAG (56:1/FA16:0)	0.0778	0.0926	0.0730	0.0448
TAG (56:1/FA18:1)	0.1067	0.1287	0.0570	0.0107
TAG (58:2/FA18:1)	0.0684	0.3709	0.0844	0.0921
DAG 12:0-12:0	0.8942	2.4602	0.4683	0.9489
DAG 12:0-14:0	0.5846	1.3283	0.3517	0.6103
DAG 12:0-14:0	0.5167	1.1779	0.3155	0.4893
DAG 12:0-16:0	0.2735	0.5035	0.2281	0.2881
DAG 12:0-18:0	0.1864	0.2604	0.1152	0.1292
DAG 12:0-18:4	0.0155	0.0170	0.0186	0.0111
DAG 12:0-20:0	0.4071	0.3397	0.2581	0.2339
DAG 12:0-22:0	0.2058	0.1558	0.1201	0.1181
DAG 12:0-22:6	1.8296	6.0857	0.8501	2.1897
DAG 14:0-14:0	0.2368	0.5114	0.1845	0.2404
DAG 14:0-14:1	0.0387	0.0921	0.0333	0.0408
DAG 14:0-16:0	0.4663	0.5802	0.5953	0.3442

PC 34:1	35.6717	35.9654	25.1749	22.1072
PC 35:1	8.6548	4.1518	4.6375	3.3550
PC 36:0	0.7888	1.7745	1.4350	0.5938
PC 36:1	8.7033	18.4476	14.7868	6.4072
PC 37:1	0.8070	1.3092	1.0815	0.5728
PC 38:1	0.6571	1.0750	0.7341	0.4796
PC 38:7	0.0451	0.0870	0.0678	0.0492
PC 39:3	0.0593	0.0981	0.0874	0.0399
PC 40:1	0.0352	0.1546	0.0623	0.0316
PC 40:8	0.1024	0.1803	0.1289	0.0910
PC 41:6	0.0132	0.0431	0.0397	0.0425
PI_26:0	0.0772	0.0750	0.0519	0.0629
PI_28:0	0.0317	0.0302	0.0208	0.0298
PI_30:2	0.0107	0.0206	0.0147	0.0123
PI_30:1	0.0027	0.0042	0.0028	0.0036
PI_30:0	0.0102	0.0090	0.0058	0.0068
PI_31:2	0.0006	0.0015	0.0009	0.0023
PI_31:1	0.0686	0.1382	0.0896	0.0701
PI_31:0	0.0052	0.0114	0.0085	0.0082
PI_32:2	0.0021	0.0043	0.0031	0.0053
PI_32:1	0.0061	0.0053	0.0057	0.0138
PI_32:0	0.0056	0.0072	0.0102	0.0252
PI_33:1	0.0081	0.0053	0.0043	0.0067
PI_33:0	0.0266	0.0041	0.0050	0.0096
PI_34:2	0.0175	0.0176	0.0144	0.0350
PI_34:1	0.0342	0.0316	0.0244	0.0953
PI_35:2	0.0073	0.0045	0.0036	0.0083
PI_35:1	0.0779	0.0104	0.0169	0.0249
PI_35:0	0.4852	0.0406	0.0702	0.1021

DAG 14:0-16:1	0.5484	0.1865	0.1435	0.2024
DAG 14:0-18:0	0.4532	0.3483	0.2820	0.2648
DAG 14:0-20:0	0.1715	0.1354	0.1119	0.0935
DAG 14:0-22:0	0.0917	0.0790	0.0519	0.0585
DAG 14:0-22:6	1.7032	4.4982	0.8569	1.5521
DAG 14:1-14:1	0.0547	0.1076	0.0429	0.0749
DAG 14:1-16:0	0.5667	0.2548	0.2199	0.4812
DAG 14:1-18:0	0.1779	0.1557	0.1214	0.1919
DAG 14:1-20:0	0.7795	0.0944	0.4995	0.1408
DAG 14:1-22:0	0.0386	0.0993	0.0496	0.0890
DAG 14:1-22:6	0.0260	0.0276	0.0190	0.0108
DAG 16:0-16:0	8.8736	7.6904	6.9023	4.8623
DAG 16:0-16:1	0.6694	1.5157	1.8644	0.6784
DAG 16:0-18:0	5.2984	4.3621	3.9562	3.3791
DAG 16:0-20:0	0.3020	0.2247	0.2519	0.2338
DAG 16:0-20:5	0.2634	0.0792	0.1699	0.1353
DAG 16:0-22:6	2.1873	3.0267	1.1945	1.4010
DAG 16:1-16:1	0.3232	0.7918	0.4949	0.4246
DAG 16:1-18:0	2.8277	1.0839	1.0636	0.7847
DAG 16:1-20:5	0.0749	0.0100	0.0451	0.0224
DAG 16:1-22:0	0.0248	0.0377	0.0351	0.0421
DAG 16:1-22:6	0.0607	0.0742	0.0518	0.0512
DAG 18:0-18:0	3.7367	3.1547	2.5277	2.2828
DAG 18:0-20:0	0.0864	0.0764	0.0505	0.0484
DAG 18:0-22:6	0.8941	1.3827	0.5520	0.7116
DAG 18:1-18:1	5.0250	5.9289	4.9352	4.0363
DAG 18:1-18:4	0.0014	0.0020	0.0009	0.0016
DAG 18:1-20:0	0.0392	0.0441	0.0281	0.0238
DAG 18:1-20:5	0.0096	0.0126	0.0091	0.0111

PI_36:2	0.0097	0.0087	0.0081	0.0177
PI_36:1	0.0119	0.0068	0.0070	0.0173
PI_38:2	0.0009	0.0010	0.0004	0.0014
PI_38:1	0.0007	0.0008	0.0008	0.0016
PI_38:0	0.0045	0.0021	0.0071	0.0041
PS_30_0	0.0019	0.0007	0.0005	0.0012
PS_32_1	0.0016	0.0016	0.0008	0.0005
PS_32_0	0.0026	0.0033	0.0025	0.0043
PS_34_1	0.0046	0.0026	0.0029	0.0025
PS_34_0	0.0008	0.0005	0.0005	0.0003
PS_35_2	0.0029	0.0005	0.0004	0.0008
PS_35_1	0.0003	0.0003	0.0002	0.0003
PS_36_1	0.0049	0.0021	0.0036	0.0022
PS_36_0	0.0007	0.0004	0.0006	0.0006

DAG 18:1-22:0	0.0308	0.0330	0.0243	0.0264
DAG 18:1-22:6	0.9633	1.3878	0.6795	0.7113
DAG 18:2-18:2	0.2364	0.3294	0.2283	0.2388
DAG 18:2-22:6	0.0886	0.1145	0.0708	0.0654
DAG 18:3-20:5	0.0103	0.0080	0.0050	0.0064
DAG 18:3-22:6	0.1677	0.1817	0.1233	0.1274
DAG 18:4-18:4	0.0186	0.0135	0.0108	0.0142
DAG 18:4-20:0	0.0128	0.0163	0.0107	0.0117
DAG 20:0-20:0	0.0180	0.0469	0.0662	0.0240
DAG 20:0-20:5	0.0479	0.0467	0.0277	0.0425
DAG 20:0-22:6	0.0673	0.0090	0.0401	0.0168
DAG 20:1-20:1	0.0107	0.0065	0.0096	0.0097
DAG 20:1-20:5	0.0335	0.0218	0.0213	0.0215
DAG 20:1-22:0	0.0171	0.0128	0.0117	0.0159
DAG 22:6-22:6	0.2161	0.0861	0.1675	0.1530

(B) CWL Extract

Analyte (GL)	DS (ng/mL)	DR (ng/mL)	MDR (ng/mL)	PXDR (ng/mL)
TAG (42:0/FA14:0)	137.76	105.18	271.00	99.52
TAG (42:0/FA16:0)	228.14	144.98	276.95	215.08
TAG (44:0/FA14:0)	80.00	58.18	140.11	74.62
TAG (44:0/FA16:0)	116.89	73.12	272.94	149.43
TAG (44:0/FA18:0)	33.85	28.71	95.72	38.23
TAG (46:0/FA14:0)	26.05	26.33	78.25	58.51
TAG (46:0/FA16:0)	61.65	50.40	161.30	112.25
TAG (46:0/FA18:0)	11.98	11.85	27.30	15.85
TAG (47:1/FA14:0)	7.35	5.83	16.45	10.75

Analyte (GPL)	DS (ng/mL)	DR (ng/mL)	MDR (ng/mL)	PXDR (ng/mL)
LPC 14:0	4.952144	8.63	9.39	48.23
LPC 15:0	4.475712	5.74	18.24	52.34
LPC 16:0	35.68899	117.70	275.57	518.12
LPC 16:1	3.72223	9.19	8.65	92.68
LPC 17:0	3.592099	4.31	15.58	18.73
LPC 18:0	10.66909	34.60	104.56	280.71
LPC 19:0	4.49241	10.54	19.71	317.63
LPC 20:0	5.275337	8.52	16.10	158.23
LPC 20:5	42.08371	133.47	165.23	692.49

TAG (47:1/FA16:0)	14.62	9.20	20.96	14.07
TAG (47:1/FA16:1)	14.73	11.72	27.10	25.11
TAG (47:1/FA17:0)	4.27	3.16	8.55	5.58
TAG (47:1/FA18:1)	5.24	5.08	17.12	12.84
TAG (48:0/FA14:0)	4.71	4.79	14.91	7.35
TAG (48:0/FA16:0)	36.11	31.17	104.88	50.62
TAG (48:0/FA18:0)	10.81	7.80	19.09	11.15
TAG (49:2/FA14:0)	1.07	0.78	3.52	1.77
TAG (49:2/FA16:0)	2.61	2.20	5.45	3.31
TAG (49:2/FA16:1)	5.86	5.32	10.29	7.46
TAG (49:2/FA17:0)	1.59	1.08	2.14	2.14
TAG (49:2/FA18:1)	3.40	3.70	13.45	3.89
TAG (49:2/FA18:2)	2.31	1.85	6.76	2.18
TAG (50:0/FA14:0)	1.06	0.78	3.47	1.54
TAG (50:0/FA16:0)	12.22	7.68	26.45	16.59
TAG (50:0/FA18:0)	7.65	5.87	24.59	9.44
TAG (52:0/FA16:0)	1.86	0.99	5.23	2.05
TAG (52:0/FA18:0)	2.82	1.59	6.79	1.30
DAG 12:0-12:0	3.72	4.52	5.20	15.32
DAG 12:0-14:0	3.16	3.10	5.03	7.91
DAG 12:0-14:0	2.64	2.45	4.01	6.92
DAG 12:0-16:0	3.05	3.86	5.39	13.41
DAG 12:0-18:0	0.76	2.00	2.72	2.20
DAG 12:0-20:0	10.03	15.36	14.54	21.73
DAG 12:0-22:0	5.23	6.13	5.87	4.28
DAG 12:0-22:6	5.62	7.78	9.57	26.51
DAG 14:0-14:0	2.59	2.84	5.33	4.45
DAG 14:0-14:1	0.37	0.25	0.50	1.74
DAG 14:0-16:0	4.09	8.78	7.84	6.00
DAG 14:0-16:1	5.55	4.82	6.55	11.87
DAG 14:0-18:0	5.32	7.33	7.30	14.15

PE 32:0	0.904898	0.78	0.97	0.46
PE 33:1	0.793245	0.56	0.86	0.42
PE 34:0	0.638235	0.49	0.41	0.39
PE 35:1	1.515165	0.72	1.01	0.67
PE 36:1	0.114639	0.06	0.08	0.15
PE 37:2	0.275227	0.14	0.07	0.13
PE 38:1	0.005807	0.01	0.01	0.01
PC 30:0	13.65771	22.92	16.87	12.28
PC 31:0	4.056006	8.68	3.99	9.77
PC 32:0	117.2597	926.33	291.06	230.28
PC 33:0	3.260626	9.90	6.31	9.39
PC 33:1	5.694216	15.30	6.43	8.46
PC 34:0	31.62033	200.98	75.97	36.12
PC 34:1	159.6527	302.46	372.65	222.25
PC 35:1	5.343692	9.71	9.04	4.02
PC 36:0	5.271176	7.93	5.92	3.19
PC 36:1	33.7071	69.63	71.76	29.36
PC 37:1	3.862982	8.73	3.64	5.07
PI_26:0	1.636202	2.39	3.81	21.90
PI_28:0	0.470085	0.75	0.90	4.85

DAG 14:0-20:0	6.02	5.57	7.81	5.52
DAG 14:0-22:0	3.57	3.02	3.39	5.94
DAG 14:0-22:6	6.06	6.25	9.63	17.76
DAG 14:1-16:0	22.82	27.33	33.44	57.96
DAG 14:1-18:0	21.51	20.65	23.78	51.85
DAG 14:1-20:0	10.02	40.49	27.21	100.79
DAG 16:0-16:0	256.04	397.20	376.54	628.06
DAG 16:0-16:1	11.16	16.58	15.01	14.25
DAG 16:0-18:0	294.01	307.70	358.27	314.83
DAG 16:0-20:0	3.57	3.83	3.62	5.01
DAG 16:0-22:6	10.02	10.45	14.61	19.80
DAG 16:1-16:1	5.53	5.94	4.20	16.15
DAG 16:1-18:0	23.18	16.20	18.98	26.31
DAG 16:1-18:4	0.21	0.25	0.26	0.72
DAG 16:1-20:0	1.39	1.42	1.63	3.97
DAG 18:0-18:0	173.39	223.91	184.49	254.22
DAG 18:0-20:0	1.40	2.78	2.11	4.61
DAG 18:0-22:0	0.57	1.32	1.35	0.52
DAG 18:0-22:6	3.03	2.99	3.06	12.17
DAG 18:1-18:1	63.91	66.46	99.24	136.65
DAG 18:1-20:0	0.60	0.56	1.11	1.93
DAG 18:1-22:0	0.37	0.36	0.28	0.80
DAG 18:1-22:6	4.95	4.49	6.21	12.71
DAG 18:2-18:2	7.23	7.81	12.08	26.28
DAG 18:2-22:6	0.34	0.58	0.46	0.66
DAG 18:3-22:0	0.15	0.32	0.47	0.70
DAG 18:3-22:6	0.87	0.71	0.74	0.77
DAG 20:0-20:0	0.21	0.54	0.37	1.18
DAG 20:0-20:5	0.65	0.72	0.71	0.87
DAG 20:0-22:0	7.42	13.12	20.59	4.09
DAG 20:1-20:5	0.44	0.59	0.44	2.81

Table S4: Percentage of differentially regulated GL (TAG and DAG) and GPL (PC, PE, LPC, PI, and PS) species derived from quantified lipids in TL and CWL extracts across DS, DR, MDR, and PXDR MTB isolates. (A) GPLs in TL extract, (B) GLs in TL extract, (C) GPLs in CWL extract, (D) GLs in CWL extract

(A) Differential regulation of GPLs in TL extract

DS vs DR			
Analyte (Lipid Species)	p-value	Down (%)	Up (%)
LPC 16:1	0.01163		65.94
LPC 17:0	0.01028	82.91	
LPC 20:5	0.00761	89.23	
LPC 22:0	0.01763	74.66	
PE 38:1	0.00041	47.22	
PC 24:0	0.00496	90.75	
PC 31:0	0.04063		47.12
PC 33:1	0.02523		69.47
PC 35:1	0.00389	52.03	
PC 36:0	0.02485		55.55
PC 36:1	0.03304		52.82
PC 38:7	0.02927		59.32
PC 40:1	0.03617		77.22
PC 40:8	0.03745		43.21
PC 41:6	0.00636		69.44
PI_31:0	0.04792		54.10
PI_33:0	0.00316	84.57	
PI_35:2	0.00587	39.15	
PI_35:1	0.00149	86.66	
PI_35:0	0.00219	91.63	

DS vs MDR			
Analyte (Lipid Species)	p-value	Down (%)	Up (%)
LPC 14:0	0.01586320	58.27	
LPC 16:1	0.00482401		77.58
PE 35:1	0.00087593	27.88	
PE 37:2	0.00006592	50.30	
PE 38:1	0.00000006	63.14	
PC 24:0	0.01178161	79.80	
PC 33:1	0.04986953		54.63
PC 35:1	0.00718223	46.42	
PC 40:1	0.01414027		43.48
PC 41:6	0.04734774		66.85
PI_28:0	0.04201690	34.52	
PI_33:1	0.04804973	46.94	
PI_33:0	0.00486248	81.06	
PI_34:2	0.03231271	17.88	
PI_34:1	0.00327406	28.78	
PI_35:2	0.00208123	50.99	
PI_35:1	0.00464234	78.25	
PI_35:0	0.00417443	85.54	
PI_36:1	0.00745387	40.74	
PI_38:2	0.01008271	50.44	

DS vs PXDR			
Analyte (Lipid Species)	p-value	Down (%)	Up (%)
LPC 15:0	0.008908	55.11	
LPC 16:0	0.042756	44.32	
LPC 16:1	0.008255		72.34
PE 32:0	0.003419	26.48	
PE 33:1	5.61E-05	33.58	
PE 34:0	0.016506	27.53	
PE 35:1	0.011414	26.03	
PE 36:1	0.028894	31.97	
PE 37:2	0.008417	47.68	
PE 38:1	3.05E-05	63.68	
PC 30:0	0.000978	41.92	
PC 31:0	0.04526		24.87
PC 32:0	0.023716	46.79	
PC 35:1	0.015705	61.24	
PC 36:1	0.047589	26.38	
PC 37:1	0.005654	29.02	
PC 38:1	0.003405	27.00	
PC 41:6	0.00108		69.04
PI_31:0	0.02227		36.13
PI_32:0	0.046927		77.60

PI_36:1	0.00712	42.93	
PI_38:0	0.03608	53.52	
PS_30_0	0.04689	61.55	
PS_34_1	0.00861	44.39	
PS_35_2	0.00059	82.90	
PS_36_1	0.03179	56.79	
PS_36_0	0.04561	47.81	

PS_30_0	0.01332519	72.61	
PS_32_1	0.00522548	48.83	
PS_34_1	0.01584341	36.26	
PS_35_2	0.00041445	85.04	
PS_35_1	0.01419860	46.83	

PI_34:2	0.006824		50.00
PI_34:1	0.038351		64.09
PI_35:1	0.046084	68.00	
PI_35:0	0.039496	78.95	
PS_32_1	0.002818	66.65	
PS_34_1	0.0435	46.48	
PS_35_2	0.020443	72.05	

(B) Differential regulation of GLs in TL extract

DS vs DR			
Analyte (Lipid Species)	p-value	Down (%)	Up (%)
Nil			

DS vs MDR			
Analyte (Lipid Species)	p-value	Down (%)	Up (%)
TAG (42:0/FA14:0)	0.00341495	56.53	
TAG (42:0/FA16:0)	0.004301654	44.90	
TAG (44:0/FA14:0)	0.009784958	45.13	
TAG (44:0/FA16:0)	0.018641758	38.96	
TAG (44:0/FA18:0)	0.044267325	33.15	
TAG (46:0/FA14:0)	0.020018516	40.62	
TAG (46:0/FA16:0)	0.017127069	40.98	
TAG (48:0/FA14:0)	0.031555968	36.66	
TAG (48:0/FA16:0)	0.005519452	48.88	
TAG (48:0/FA18:0)	0.039408803	35.26	
TAG (50:0/FA14:0)	0.038382014	36.19	
TAG (50:0/FA16:0)	0.008680686	43.08	
TAG (50:0/FA18:0)	0.018568501	38.92	
TAG (52:0/FA16:0)	0.01072697	41.77	
TAG (52:0/FA18:0)	0.025066637	36.18	

DS vs PXDR			
Analyte (Lipid Species)	p-value	Down (%)	Up (%)
TAG (42:0/FA14:0)	0.017434	60.09	
TAG (42:0/FA16:0)	0.000377	65.43	
TAG (44:0/FA14:0)	0.0009	66.32	
TAG (44:0/FA16:0)	3.07E-05	70.33	
TAG (44:0/FA18:0)	2.3E-06	69.53	
TAG (46:0/FA14:0)	0.000114	70.10	
TAG (46:0/FA16:0)	8.7E-05	69.28	
TAG (46:0/FA18:0)	4.95E-09	71.83	
TAG (47:1/FA14:0)	0.000507	39.12	
TAG (47:1/FA16:0)	0.00045	36.33	
TAG (47:1/FA16:1)	0.00045	30.23	
TAG (47:1/FA17:0)	6.57E-06	42.29	
TAG (47:1/FA18:1)	0.000275	47.28	
TAG (48:0/FA14:0)	2.25E-06	75.17	
TAG (48:0/FA16:0)	0.00049	73.52	

TAG (52:0/FA20:0)	0.005716725	57.78	
TAG (54:0/FA16:0)	0.026253817	69.56	
DAG 12:0-12:0	0.008444931	47.63	
DAG 12:0-14:0	0.022244874	39.83	
DAG 12:0-14:1	0.024652037	38.93	
DAG 12:0-20:0	0.028966644	36.61	
DAG 12:0-22:0	0.001350509	41.63	
DAG 12:0-22:6	0.011201189	53.54	
DAG 14:0-16:1	0.00218948	73.83	
DAG 14:0-18:0	0.027405335	37.77	
DAG 14:0-20:0	0.009002445	34.72	
DAG 14:0-22:0	0.00013632	43.33	
DAG 14:0-22:6	0.008449322	49.69	
DAG 14:1-16:0	0.001014361	61.19	
DAG 16:0-18:0	0.014204933	25.33	
DAG 16:0-22:6	0.01192698	45.39	
DAG 16:1-18:0	0.001777596	62.39	
DAG 16:1-20:0	0.003874748	91.01	
DAG 18:0-18:0	0.001817883	32.36	
DAG 18:0-20:0	0.003067727	41.55	
DAG 18:0-22:6	0.036479982	38.27	
DAG 18:3-20:5	0.001822435	50.84	
DAG 18:4-18:4	0.001830814	41.88	
DAG 20:0-20:5	0.001529566	42.23	
DAG 20:1-20:5	0.01759621	36.54	

TAG (48:0/FA18:0)	1.55E-06	74.67	
TAG (49:2/FA14:0)	0.005023	35.81	
TAG (49:2/FA16:0)	0.005998	37.59	
TAG (49:2/FA16:1)	5.55E-06	39.46	
TAG (49:2/FA17:0)	5.02E-06	51.18	
TAG (49:2/FA18:1)	5.34E-09	50.49	
TAG (49:2/FA18:2)	0.003675	49.33	
TAG (50:0/FA14:0)	1.8E-07	77.10	
TAG (50:0/FA16:0)	1.09E-05	76.78	
TAG (50:0/FA18:0)	1.13E-06	77.12	
TAG (51:0/FA16:0)	0.000965	61.26	
TAG (51:0/FA17:0)	0.000679	65.36	
TAG (51:0/FA18:0)	2.23E-07	77.17	
TAG (51:3/FA16:1)	3.54E-06	52.73	
TAG (51:3/FA17:0)	0.022668	56.35	
TAG (52:0/FA16:0)	1.66E-05	78.07	
TAG (52:0/FA18:0)	9.5E-06	78.49	
TAG (52:0/FA20:0)	0.001937	77.77	
TAG (54:0/FA18:0)	0.017473	84.04	
TAG (55:7/FA22:6)	0.007639	68.65	
TAG (56:1/FA18:1)	0.01284	90.01	
DAG 12:0-20:0	0.002569	42.54	
DAG 12:0-22:0	0.004749	42.62	
DAG 14:0-16:0	0.020514	26.17	
DAG 14:0-16:1	0.037926	63.09	
DAG 14:0-18:0	0.001172	41.56	
DAG 14:0-20:0	0.002619	45.45	
DAG 14:0-22:0	0.018001	36.21	
DAG 14:1-20:0	2.09E-05	81.93	

DAG 14:1-22:0	0.005779		56.61
DAG 14:1-22:6	0.000289	58.64	
DAG 16:0-16:0	0.000853	45.21	
DAG 16:0-18:0	0.004229	36.23	
DAG 16:0-22:6	0.01189	35.95	
DAG 16:1-18:0	0.002464	72.25	
DAG 16:1-20:0	0.033854	88.03	
DAG 18:0-18:0	0.002481	38.91	
DAG 18:0-20:0	0.014823	44.05	
DAG 18:1-20:0	0.00968	39.22	
DAG 20:1-20:5	0.03731	35.93	

(C) Differential regulation of GPLs in CWL extract

DS vs DR			
**Analyte (Lipid Species)**	**p-value**	**Down (%)**	**Up (%)**
LPC 16:0	0.00363		69.68
LPC 18:0	0.03688		70.57
PE 33:1	0.04328	29.85	
PE 35:1	0.00048	52.67	
PE 37:2	0.00372	50.14	
PC 33:0	0.03586		67.07
PC 34:0	0.04514		84.27

DS vs MDR			
**Analyte (Lipid Species)**	**p-value**	**Down (%)**	**Up (%)**
LPC 17:0	0.03826		76.95
LPC 20:0	0.01791		67.23
LPC 20:5	0.01463		74.53
PE 37:2	0.00003	77.08	
PC 32:0	0.00606		59.71
PC 34:0	0.01435		58.38

DS vs PXDR			
**Analyte (Lipid Species)**	**p-value**	**Down (%)**	**Up (%)**
LPC 17:0	0.03150		80.82
PE 32:0	0.01033	49.62	
PE 33:1	0.00782	47.35	
PE 34:0	0.04029	39.47	
PE 35:1	0.00191	55.69	
PE 37:2	0.01299	52.27	
PE 38:1	0.03264		54.79

(D) Differential regulation of GLs in CWL extract

DS vs DR			
Analyte (Lipid Species)	p-value	Down (%)	Up (%)
DAG 12:0-18:0	0.01158		61.89993
DAG 12:0-20:0	0.02557		34.68241
DAG 14:0-14:1	0.02695	31.563	
DAG 14:0-16:0	0.00115		53.43736
DAG 16:0-16:0	0.03293		35.53882
DAG 18:3-22:0	0.04507		71.35285

DS vs MDR			
Analyte (Lipid Species)	p-value	Down (%)	Up (%)
DAG 12:0-16:0	0.00433		42.42
DAG 12:0-18:0	0.00429		58.30
DAG 14:0-16:0	0.03660		44.34
DAG 14:0-18:0	0.04838		28.35
DAG 14:1-20:0	0.02975		52.08
DAG 16:0-16:0	0.01508		30.57
DAG 18:1-22:0	0.03746	80.12	

DS vs PXDR			
Analyte (Lipid Species)	p-value	Down (%)	Up (%)
DAG 12:0-16:0	0.02886		80.98
DAG 12:0-20:0	0.00321		61.24
DAG 12:0-22:6	0.02029		82.21
DAG 14:0-14:0	0.03576		51.41
DAG 14:0-16:0	0.00979		38.46
DAG 14:0-16:1	0.01967		58.36
DAG 14:0-18:0	0.00349		67.20
DAG 14:1-18:0	0.03503		64.91
DAG 16:0-16:0	0.01821		64.79
DAG 16:0-20:0	0.04574		39.81
DAG 18:0-18:0	0.02985		42.53
DAG 18:3-22:0	0.02695		87.79
DAG 20:1-20:5	0.04456		86.85

Figure. S.1 : Multivariate and statistical analysis of merged TL and CWL IDA data extracted from DS, DR, MDR, and PXDR MTB clinical isolates. (A) Radar plot representing the total number of queried m/z values in red and the number of identified m/z values (green) across all isolates. (B) PCA (top penal) and PLS-DA (bottom penal) plots showing partial overlap between resistance MTB isolates, with limited group segregation. (C) One-way ANOVA plot illustrating the significance and fold-change of differentially abundant lipid categories. (D) Venn diagram exhibiting the distribution of unique and shared lipid molecules among DS, DR, MDR, and PXDR isolates; most lipids are conserved, but each resistance phenotype also has unique lipid features. (E) Hierarchical clustering dendrogram indicating partial segregation among all referred MTB isolates, DS in green color, DR in red color, MDR in blue color, and PXDR in sky blue.

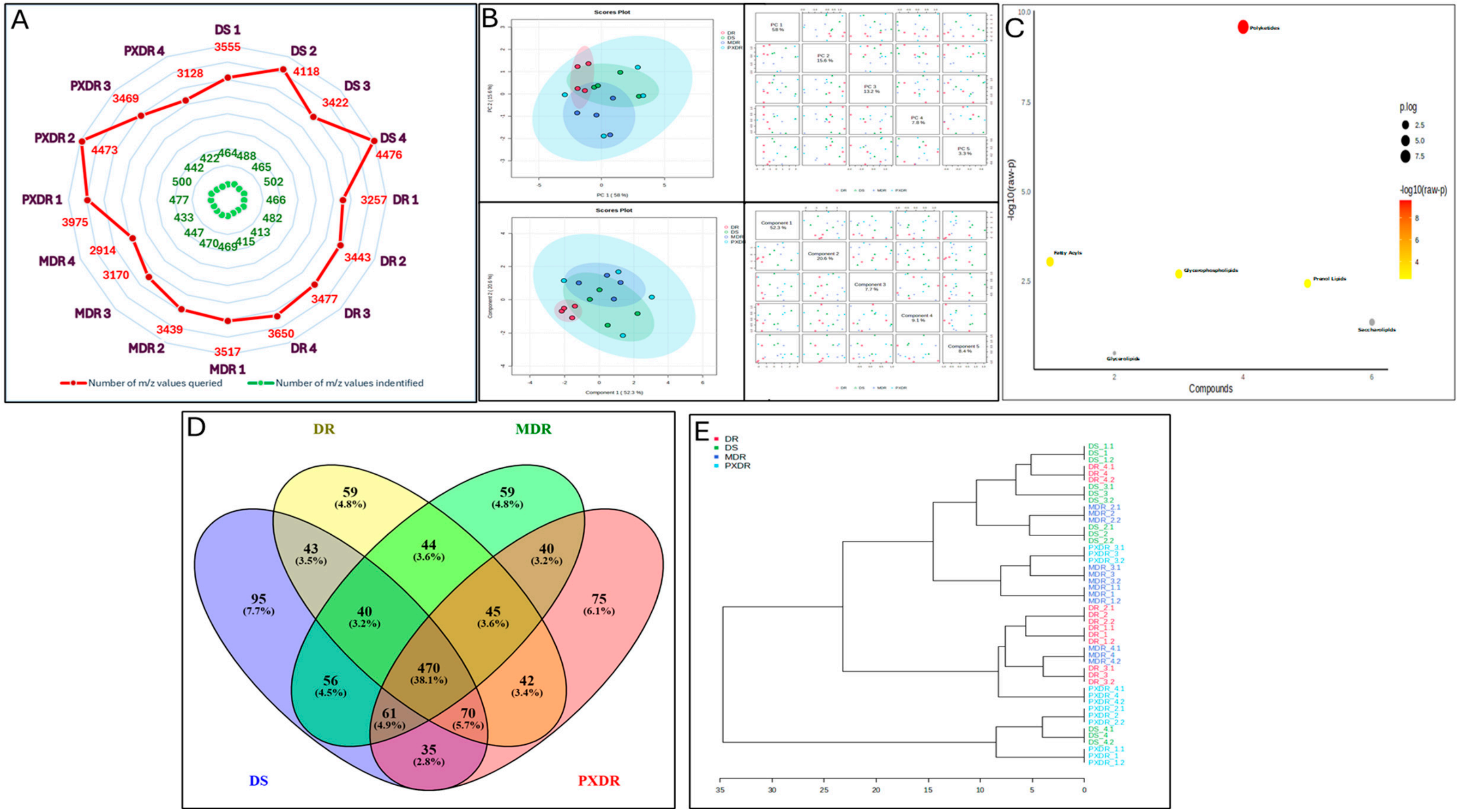

Figure. S.2 IDA Chromatograms of isolates in Pos & Neg ionization mode. (A) and (B) Pos and Neg chromatograms of TL extract, (C) and (D) Pos and Neg chromatograms of CWL extract.

(A) TL_Pos

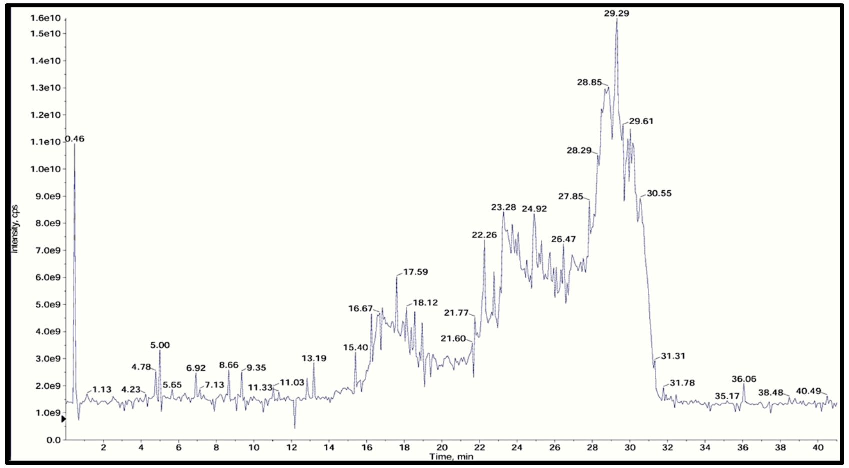

(B) TL_Neg

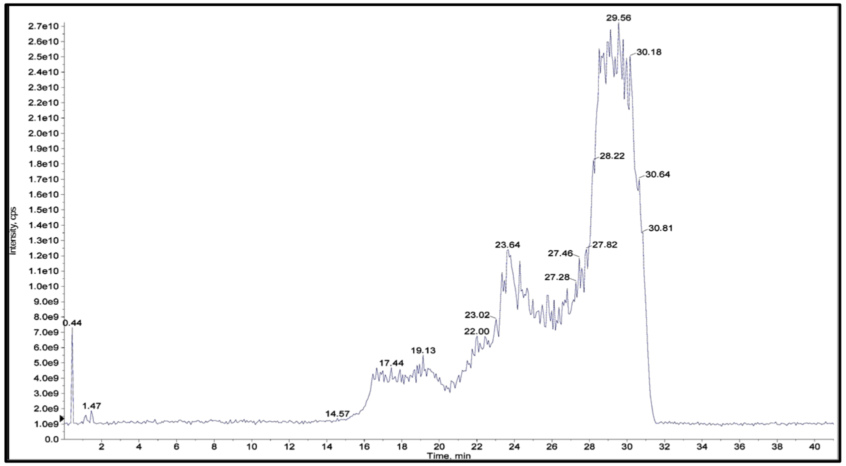

(C) CWL_Pos

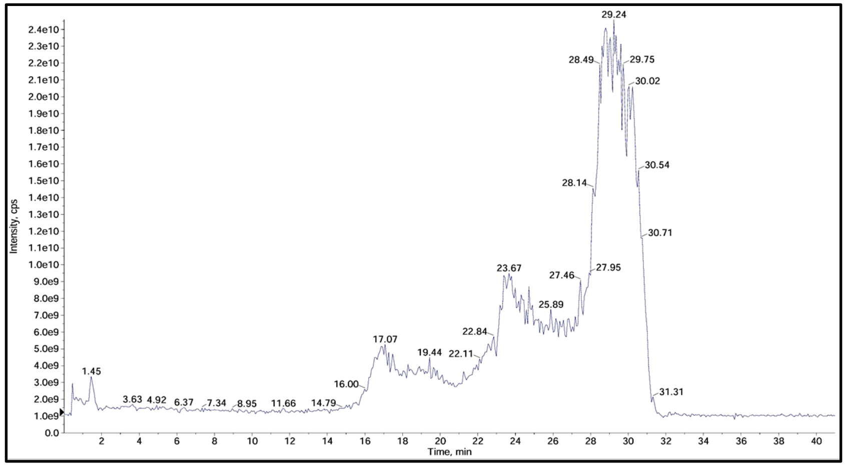

(D) CWL_Neg

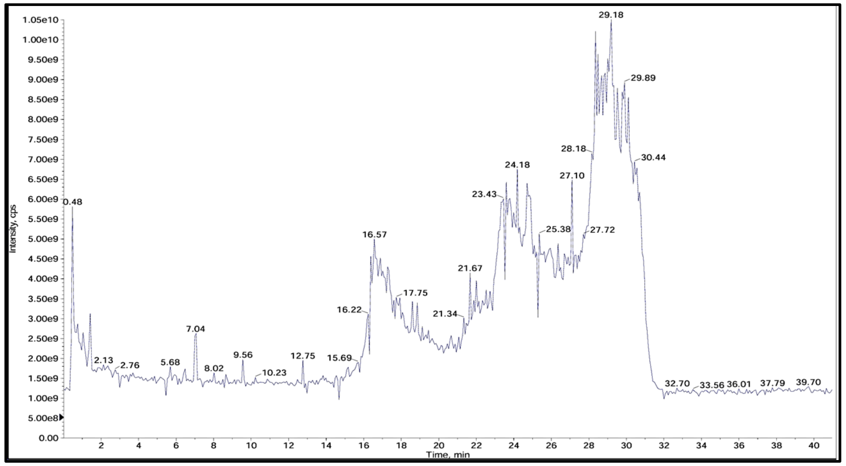

Figure. S.3 Lipid class-based PCA analysis of TL and CWL extracts across DS, DR, MDR, and PXDR MTB isolates showing phenotype-associated clustering trends at the lipid category level. (A) Lipid class-based PCA for TL extracts; (B) Lipid class-based PCA for CWL extracts

(A) Lipid class-based PCA for TL extracts

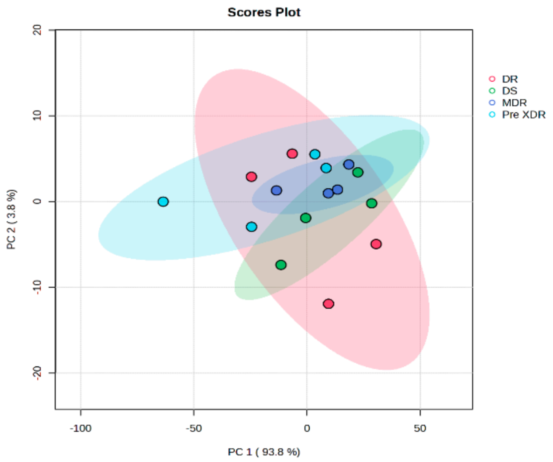

(a) Fatty Acyls

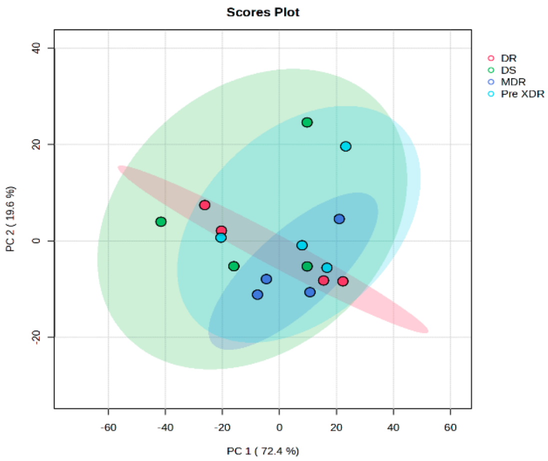

(b) Glycerolipids

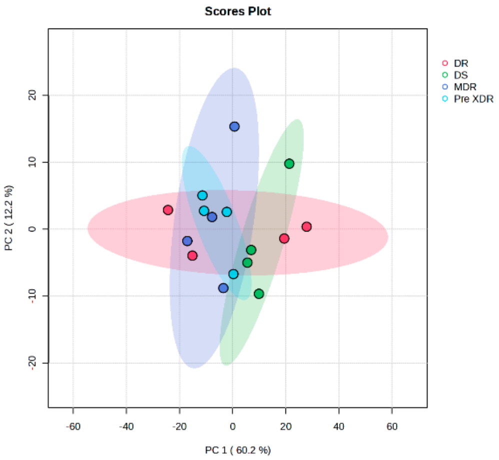

(c) Glycerophospholipids

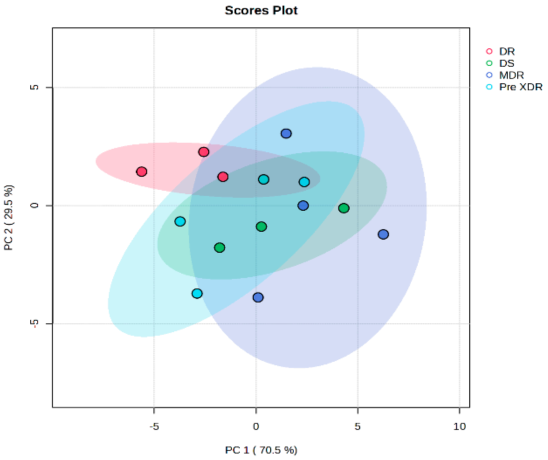

(d) Polyketides

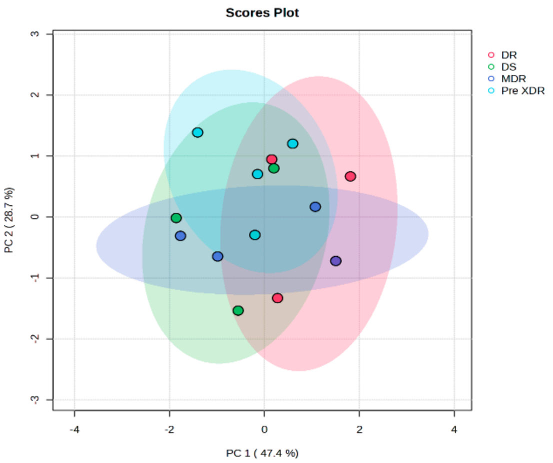

(e) Prenol Lipids

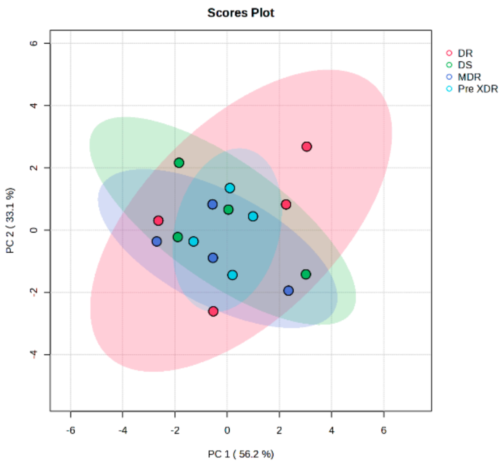

(f) Saccharolipids

(B) Lipid-class based PCA for CWL extracts

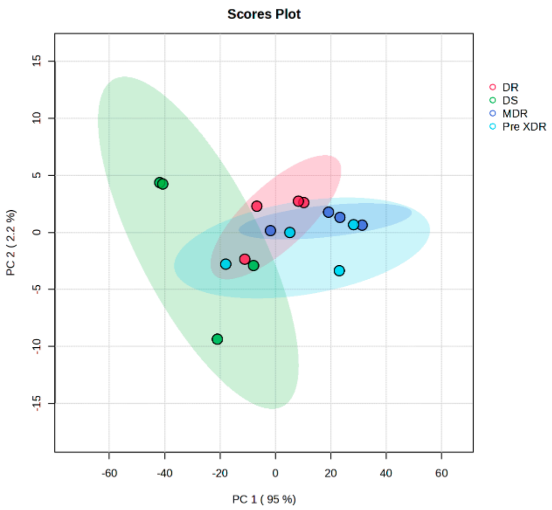

(a) Fatty Acyls

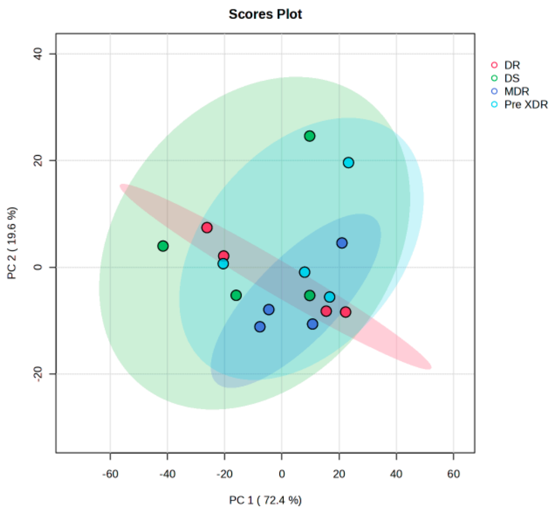

(b) Glycerolipids

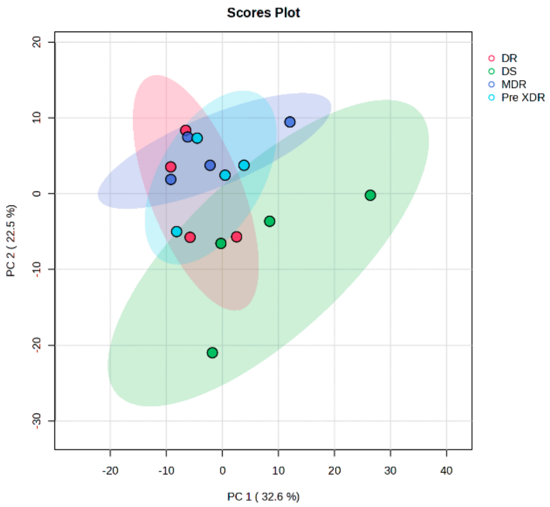

(c) Glycerophospholipids

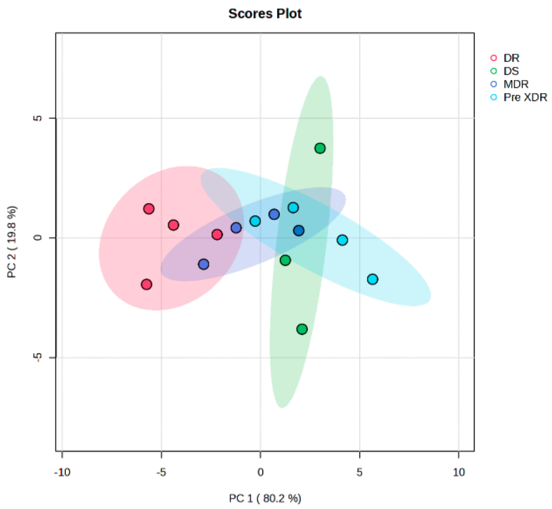

(d) Polyketides

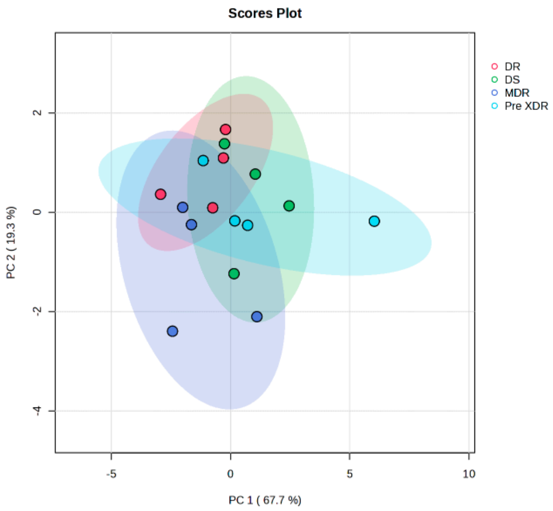

(e) Prenol Lipids

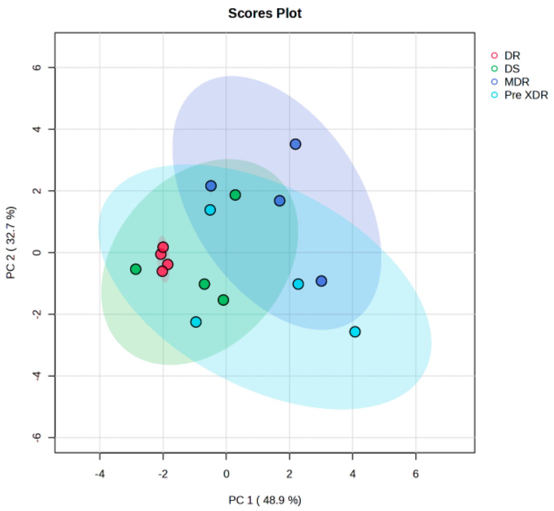

(f) Saccharolipids

Figure. S.4 XIC Chromatograms of GL (TG & DG) and GPL (PC, LPC, PE, PI, & PS) categories acquired from targeted mass spectrometry. (A) Chromatograms of TL extract, (B) chromatograms of CWL extract.

(A) Chromatograms of TL extract

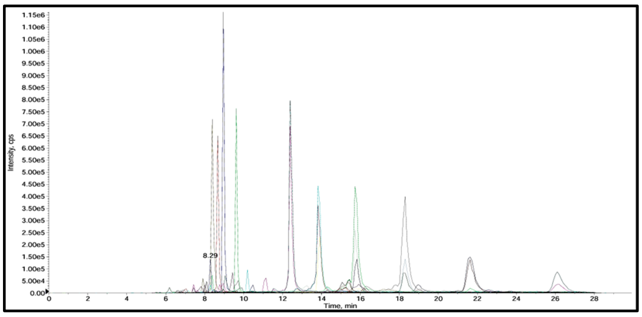

(a) GL

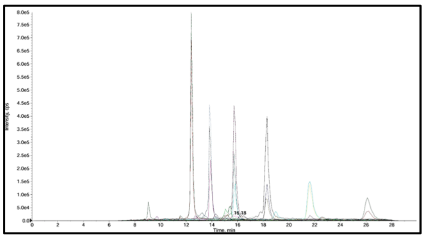

(a1) TAG

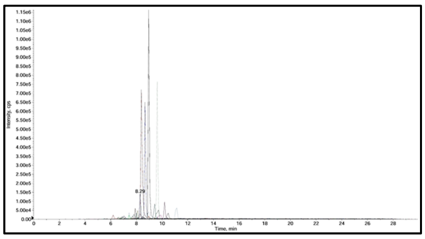

(a2) DAG

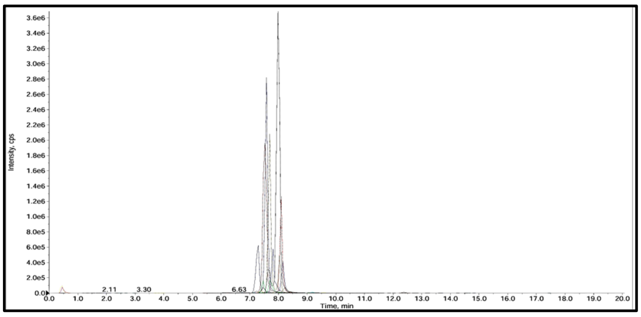

(b) GPL

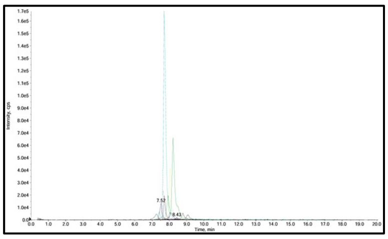

(b1) LPC

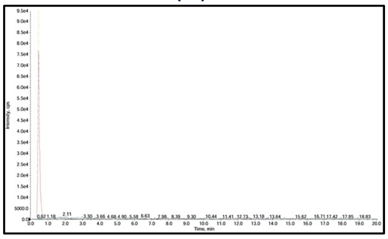

(b2) PC

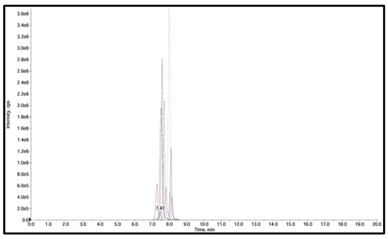

(b3) PE

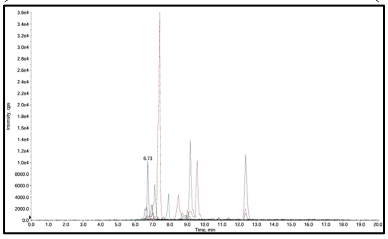

(b4) PI

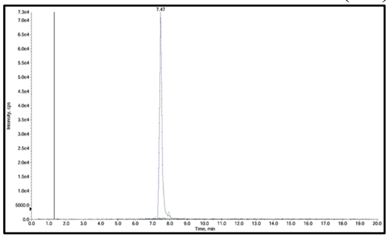

(b5) PS

(B) Chromatograms of CWL extract

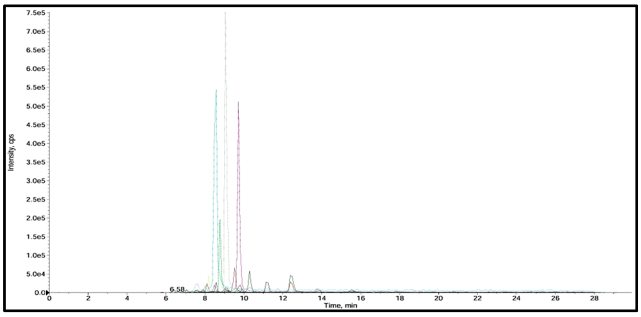

(a) GL

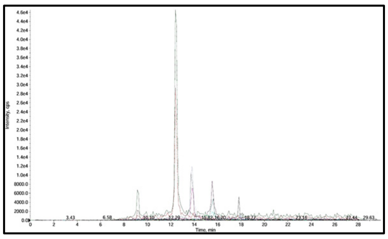

(a1) TAG

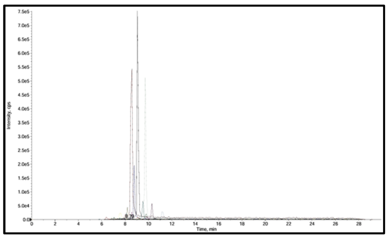

(a2) DAG

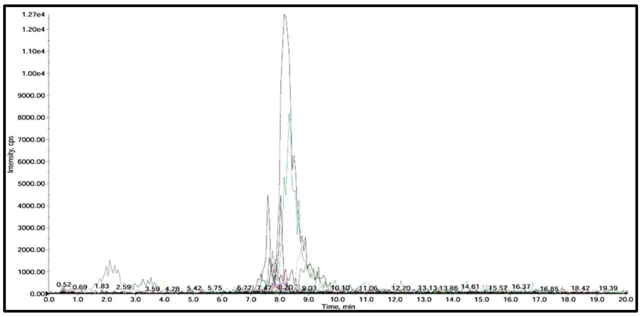

(b) GPL

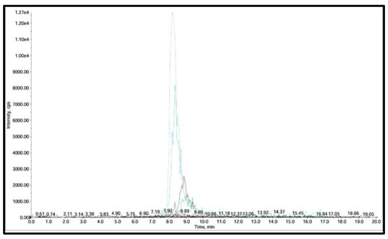

(b1) LPC

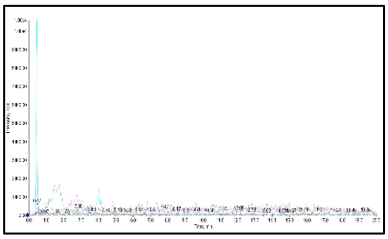

(b2) PC

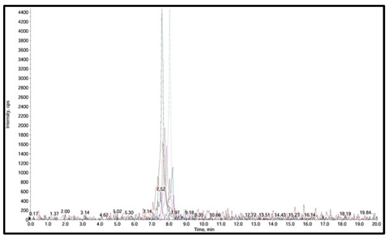

(b3) PE

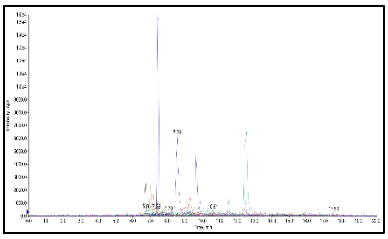

(b4) PI

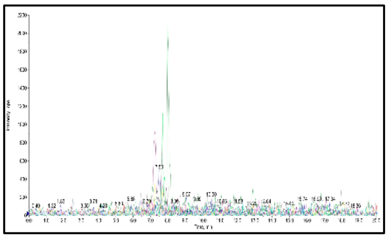

(b5) PS

Figure. S.5 : Box plots of potential lipid biomarkers identified in TL and CWL extracts of all referred MTB clinical isolates. (A) TL data highlights 2 FAs, 9 GLs, 6 GPLs, and 1 PR lipid species as potential biomarkers; (B) CWL data reveals 6 FAs, 10 GLs, 16 GPLs, 1 PK, and 1 PR lipid species as potential biomarkers.

A.

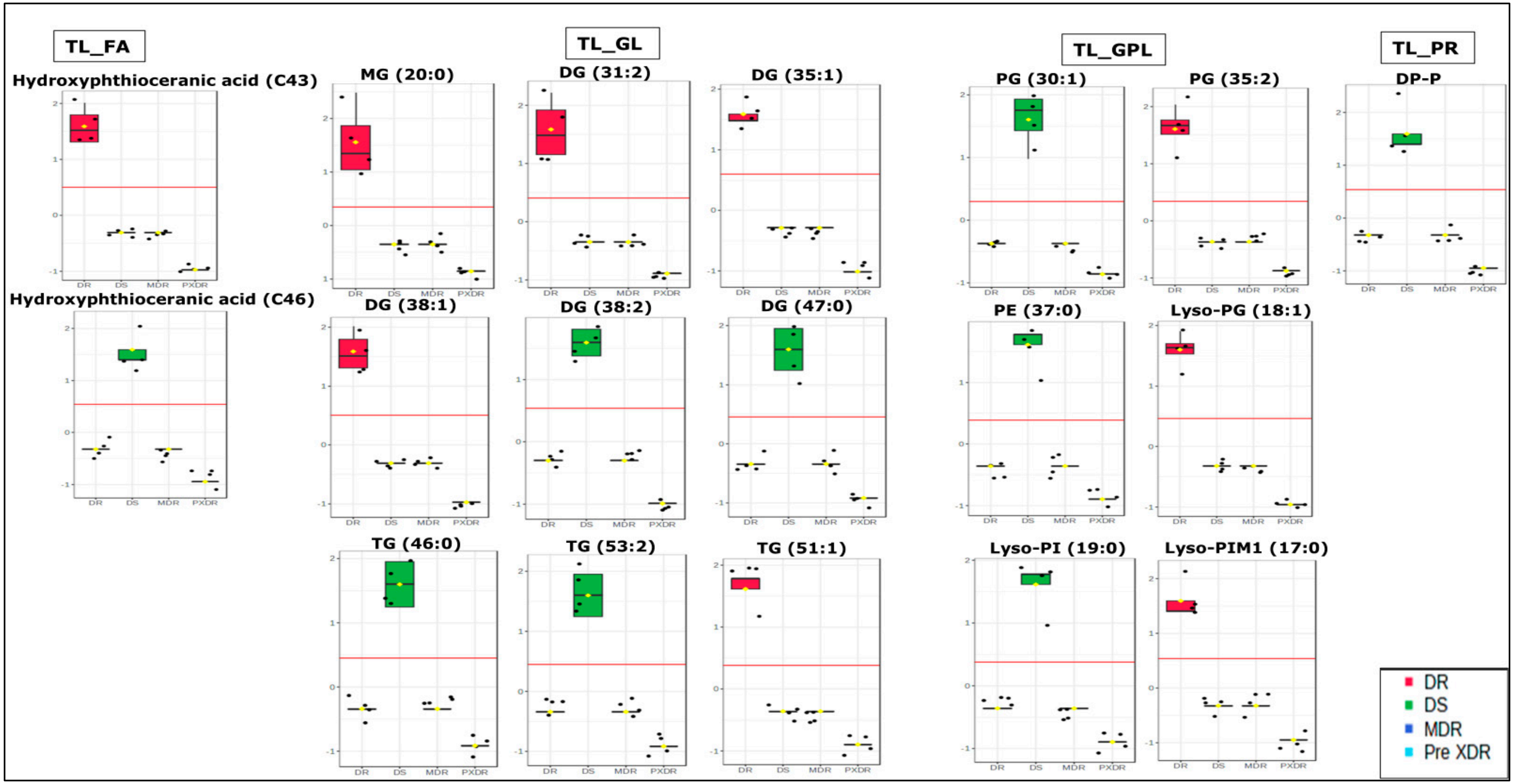

B.

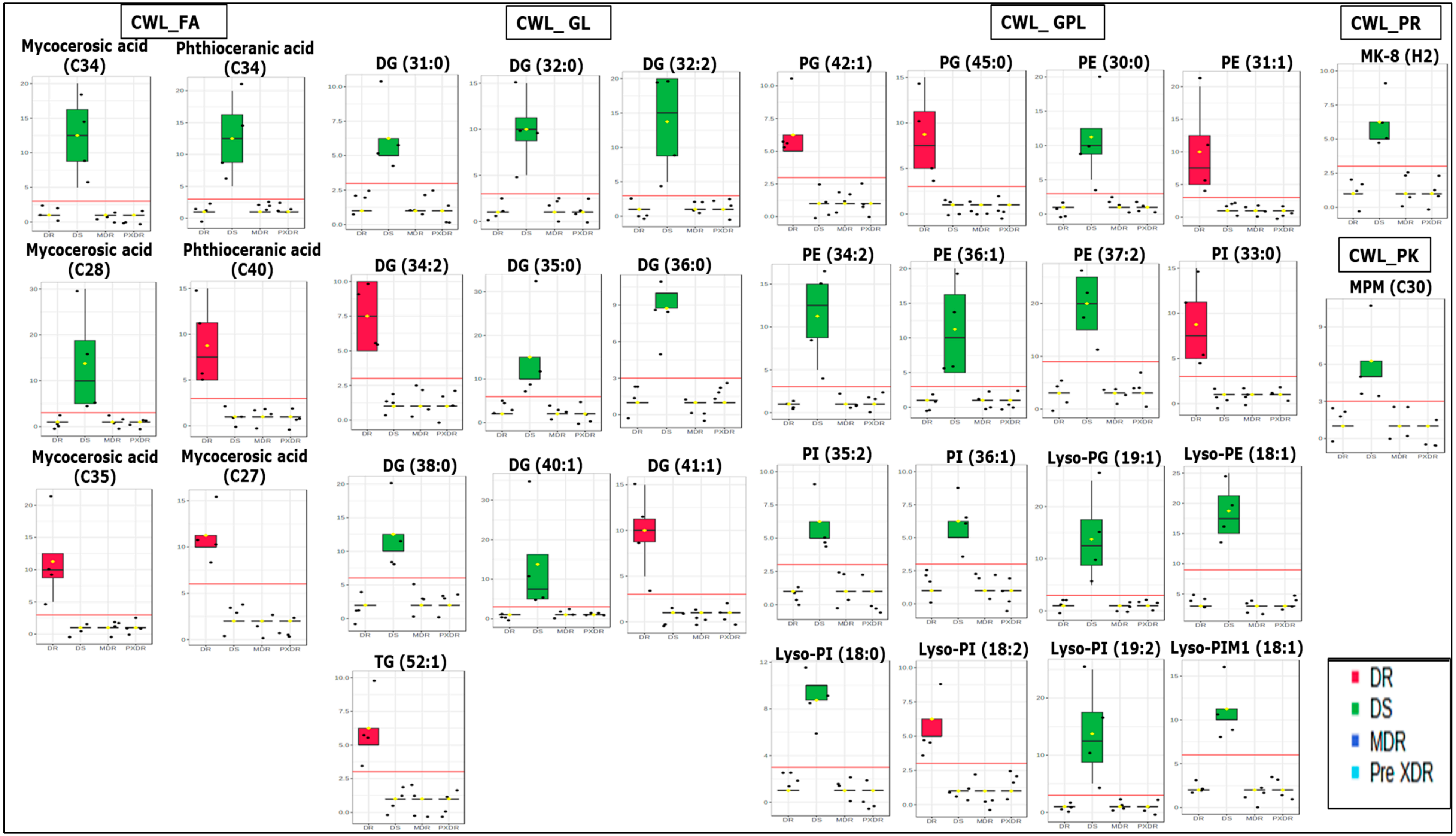
CWL_FA
Mycocerosic acid (C34)
Phthioceranic acid (C34)
Mycocerosic acid (C28)
Phthioceranic acid (C40)
Mycocerosic acid (C35)
Mycocerosic acid (C27)
CWL_ GL
DG (31:0)
DG (32:0)
DG (32:2)
DG (34:2)
DG (35:0)
DG (36:0)
DG (38:0)
DG (40:1)
DG (41:1)
TG (52:1)
CWL_ GPL
PG (42:1)
PG (45:0)
PE (30:0)
PE (31:1)
PE (34:2)
PE (36:1)
PE (37:2)
PI (33:0)
PI (35:2)
PI (36:1)
Lyso-PG (19:1)
Lyso-PE (18:1)
Lyso-PI (18:0)
Lyso-PI (18:2)
Lyso-PI (19:2)
Lyso-PIM1 (18:1)
CWL_PR
MK-8 (H2)
CWL_PK
MPM (C30)
DR
DS
MDR
Pre XDR
